# Integrative Molecular Profiling of miR-548f-3p in Triple-Negative Breast Cancer Highlights *ANP32E* as a Candidate Downstream Effector

**DOI:** 10.3390/ijms27177589

**Published:** 2026-08-25

**Authors:** Samira Behroozi, Mahdieh Salimi, Hossein Lanjanian, Najaf Allahyari Fard, Mahsa Torkamanian-Afshar, Mitra Ataei

**Affiliations:** 1 Department of Medical Genetics, Institute of Medical Biotechnology, National Institute of Genetic Engineering and Biotechnology (NIGEB), Tehran 1497716316, Iran; s_behrouzi@nigeb.ac.ir (S.B.); mit.ataei@gmail.com (M.A.); 2 Cellular and Molecular Endocrine Research Center, Research Institute for Endocrine Molecular Biology, Research Institute for Endocrine Sciences, Shahid Beheshti University of Medical Sciences, Tehran 1985717413, Iran; h.lanjanian@ut.ac.ir; 3 Department of Systems Biotechnology, National Institute of Genetic Engineering and Biotechnology (NIGEB), Tehran 1497716316, Iran; allahyar@nigeb.ac.ir; 4 Software Engineering Department, Engineering Faculty, Istanbul Topkapi University, 34087 Istanbul, Türkiye; mahsatorkamanianafshar@topkapi.edu.tr

**Keywords:** triple-negative breast cancer, miR-548f-3p, *ANP32E*, microRNA therapeutics, post-transcriptional regulation, epigenetic regulation, chromatin remodeling

## Abstract

Triple-negative breast cancer (TNBC) remains a major therapeutic challenge due to pronounced molecular heterogeneity, transcriptional plasticity, and frequent treatment resistance. MicroRNA (miRNA)-based strategies have emerged as potential approaches for modulating dysregulated gene expression networks in TNBC; however, the contribution of understudied miRNA families to TNBC-associated regulatory programs remains incompletely understood. This study aimed to investigate the tumor-suppressive role of miR-548f-3p in TNBC and to identify candidate downstream effectors, with particular focus on ANP32E. An integrative analysis combining public transcriptomic datasets, clinical expression profiling, computational target prediction, network-based prioritization, pathway analysis, and single-cell transcriptomic assessment identified miR-548f-3p as consistently downregulated in TNBC. Among candidate downstream targets, *ANP32E*, a chromatin-associated regulator involved in H2A.Z histone variant dynamics, was identified as a potential effector exhibiting increased expression in TNBC and enrichment within malignant epithelial cell populations. An inverse association between miR-548f-3p and *ANP32E* expression was observed in patient-derived samples. In breast cancer cell models, miR-548f-3p mimic restoration increased apoptosis, promoted G_0_/G_1_ accumulation, and reduced migration- and invasion-associated readouts, although measurable effects were also observed in non-tumorigenic MCF-10A cells. These phenotypic changes were accompanied by reduced *ANP32E* expression at the protein level, indicating that ANP32E expression is responsive to miR-548f-3p restoration. This study supports miR-548f-3p as a candidate tumor-suppressive miRNA in TNBC. The reduction in *ANP32E* protein expression following miR-548f-3p restoration, together with computational, single-cell, and clinical expression evidence, supports *ANP32E* as an expression-responsive candidate downstream effector of miR-548f-3p. Further reporter-based and rescue experiments are required to confirm direct 3′UTR-mediated targeting and to define the mechanistic contribution of *ANP32E* within the broader miR-548f-3p regulatory network.

## 1. Introduction

Breast cancer remains the most commonly diagnosed cancer among women worldwide. According to the World Health Organization (WHO), approximately 2.4 million women were diagnosed with breast cancer globally in 2024, and an estimated 694,000 deaths were attributed to the disease. Recent projections from the International Agency for Research on Cancer (IARC) further indicate that, if current incidence and mortality rates continue, the annual global burden could reach approximately 3.2 million new breast cancer cases and 1.1 million breast cancer-related deaths by 2050. These trends underscore the growing global health burden of breast cancer and the need for improved molecularly informed diagnostic and therapeutic strategies [1,2]. Within this heterogeneous disease, breast tumors lacking estrogen receptor (ER), progesterone receptor (PR), and human epidermal growth factor receptor 2 (HER2) expression represent a biologically diverse group of malignancies characterized by extensive transcriptional heterogeneity, lineage plasticity, immune evasion, and early therapeutic resistance [3]. Among these tumors, triple-negative breast cancer (TNBC) is associated with aggressive clinical behavior, rapid recurrence, and disproportionately poor outcomes compared with other breast cancer subtypes [4].

The absence of broadly actionable receptors, together with dynamic rewiring of oncogenic and stress-adaptive transcriptional programs, continues to limit the durability of current therapeutic strategies in this disease. Recent therapeutic advances, including immune checkpoint blockade and antibody–drug conjugates, have improved outcomes in selected TNBC settings [5]. Nevertheless, clinical benefit remains constrained by molecular heterogeneity, variable biomarker-defined responsiveness, acquired resistance, and disease relapse. These limitations underscore the need for therapeutic approaches capable of modulating the regulatory networks that sustain TNBC progression rather than targeting single surface receptors or isolated signaling nodes.

Particularly, RNA-based approaches such as miRNA- and siRNA-driven modalities have gained increasing attention for their ability to modulate gene expression with high specificity and adaptability. Such strategies offer a promising avenue to overcome conventional therapeutic barriers and address the complex regulatory networks that drive TNBC progression and treatment resistance [6]. miRNAs act as master post-transcriptional regulators; however, how specific miRNA-mediated circuits are integrated into broader epigenetic regulatory networks in TNBC remains incompletely understood [7,8]. This regulatory capacity has driven growing translational efforts toward miRNA-based therapeutic modulation, including miRNA mimics and anti-oncomiR strategies [9].

Collectively, these findings highlight miRNA-based approaches as promising next-generation therapeutic strategies for otherwise refractory cancers [10]. Notably, preclinical studies have demonstrated that systemic delivery of miRNA mimics, such as miR-22-3p, suppresses TNBC growth in orthotopic models, thereby supporting the translational feasibility of miRNA replacement therapy in this subtype [11]. In this context, miRNA mimic therapy emerges as a particularly attractive strategy for restoring disrupted regulatory networks in tumors with high transcriptional plasticity, including TNBC [12].

The miR-548 family represents one of the largest miRNA families in the human genome and has been increasingly implicated in cancer-associated regulatory processes. However, despite the reported involvement of several miR-548 family members in different malignancies, miR-548f-3p remains comparatively understudied, particularly in breast cancer and TNBC [13,14]. This study aimed to characterize the potential tumor-suppressive role of miR-548f-3p in TNBC and to evaluate ANP32E as a candidate downstream effector.

## 2. Results

### 2.1. In Silico Analyses

#### 2.1.1. miR-548 Family Screening Identifies miR-548f-3p as a Downregulated Candidate in TNBC

**Figure 1:**
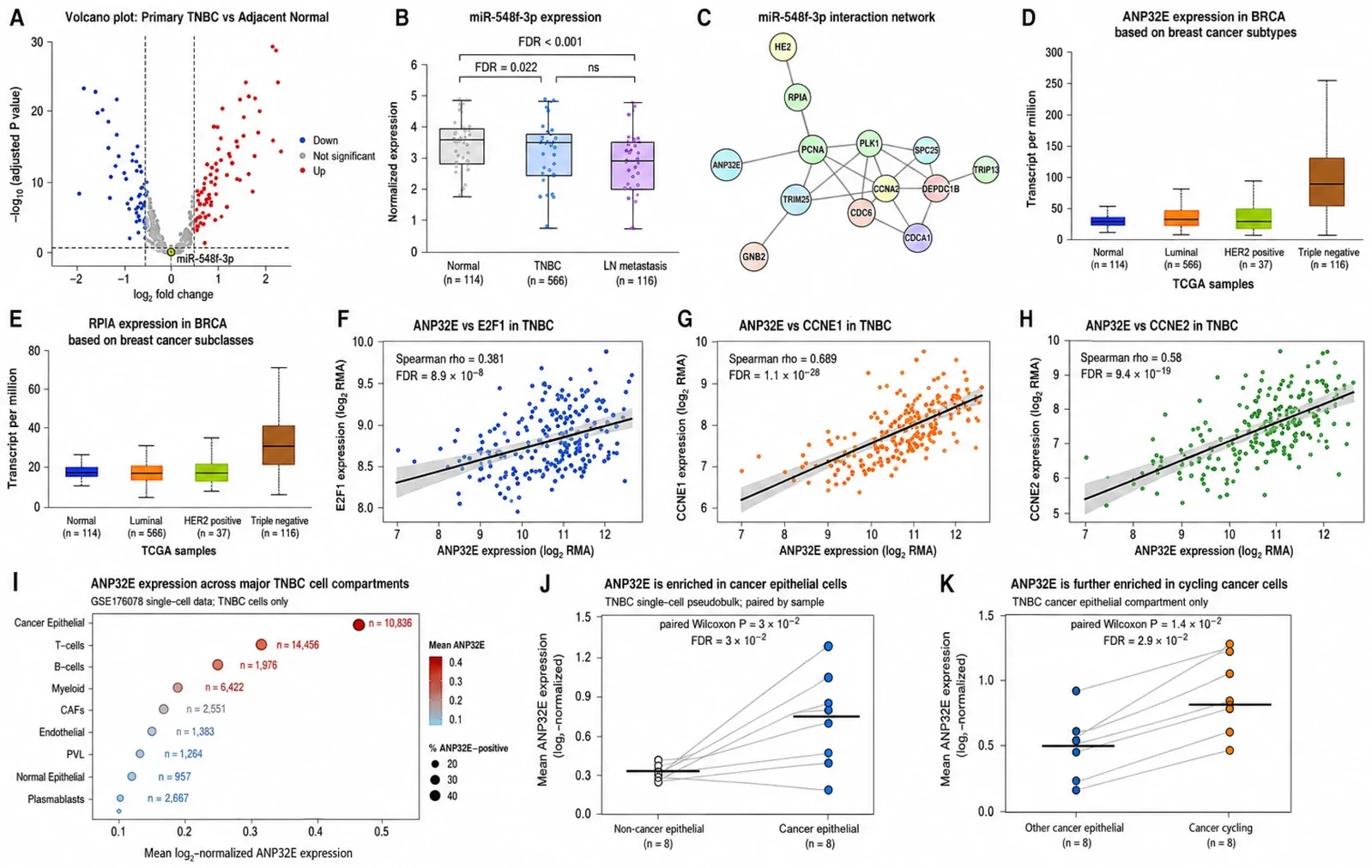
Integrated computational and transcriptomic analysis of *ANP32E* as a candidate downstream effector of miR-548f-3p in breast cancer. (**A**) Volcano plot showing the global distribution of differentially expressed miRNAs in GSE45498 comparing primary TNBC tumors with adjacent normal breast tissues. (**B**) Boxplot showing miR-548f-3p expression across adjacent normal tissues, primary TNBC tumors, and lymph node metastatic samples. (**C**) STRING-based protein–protein interaction network of prioritized candidate genes with predicted miR-548f-3p target support and TNBC-associated upregulation. (**D**,**E**) TCGA-based subtype expression analysis of *ANP32E* and *RPIA* across normal breast tissue, luminal, HER2-positive, and triple-negative breast cancer samples. (**F**–**H**) Spearman correlation analysis between *ANP32E* and representative *E2F*/*CCNE* cell-cycle regulatory genes, including *E2F1*, *CCNE1*, and *CCNE2*, in TNBC samples. (**I**) Single-cell RNA-seq analysis showing *ANP32E* expression across major TNBC cell compartments. (**J**) Paired single-cell comparison of *ANP32E* expression between non-cancer epithelial and cancer epithelial compartments. (**K**) *ANP32E* expression was compared between Cancer Cycling cells and the remaining Cancer Epithelial cells in matched TNBC samples. Statistical significance was assessed using a paired Wilcoxon signed-rank test with Benjamini–Hochberg FDR correction (*p* = 0.014, FDR = 0.029). Exact *p* values or FDR-adjusted values are shown in the corresponding panels; ns indicates not significant. In panel A, blue, red, and gray points indicate downregulated, upregulated, and non-significant miRNAs, respectively. In panel I, point color represents mean *ANP32E* expression and point size represents the percentage of ANP32E-positive cells.

The miR-548 family was selected a priori for focused investigation based on previous evidence implicating several of its members in cancer-associated regulatory processes. Given the limited functional characterization of individual miR-548 family members in triple-negative breast cancer (TNBC), we examined the expression profile of this family in the GEO dataset GSE45498. In the global differential-expression analysis of GSE45498, miRNAs meeting both |log_2_FC| ≥ 0.5 and an FDR-adjusted *p* value < 0.05 were considered formally differentially expressed. Within the miR-548 family, hsa-miR-548g and hsa-miR-548a-5p satisfied these global criteria. Because the miR-548 family had been pre-specified for focused investigation, a secondary family-level screen was also performed in which downregulated miR-548 members with an FDR-adjusted *p* value < 0.05 were retained for further consideration irrespective of the global fold-change cutoff. Seven miR-548 family members showed FDR-significant downregulation in this focused analysis (Table 1), while the overall distribution of differentially expressed miRNAs in the primary TNBC-versus-adjacent-normal comparison is shown in Figure 1A.

Among these family-focused candidates, miR-548f-3p showed FDR-significant downregulation in TNBC relative to adjacent normal tissue (log_2_FC = −0.3116; FDR-adjusted *p* = 0.02163). Although miR-548f-3p did not meet the global |log_2_FC| ≥ 0.5 threshold and was therefore not classified as a formally differentially expressed miRNA in the global analysis, it was retained for downstream investigation because of its FDR-supported downregulation and comparatively limited characterization in breast cancer, particularly TNBC. Accordingly, miR-548f-3p was prioritized as a hypothesis-driven candidate within the pre-specified miR-548 family rather than on the basis of fold-change ranking alone. Although the primary comparison in this study was TNBC tumors versus adjacent normal breast tissues, miR-548f-3p expression was also evaluated in lymph node metastatic samples available in GSE45498. miR-548f-3p expression was significantly lower in primary TNBC tumors than in adjacent normal tissues and showed a further reduction in lymph node metastatic samples compared with adjacent normal tissues. However, the difference between primary TNBC and lymph node metastatic samples was not statistically significant. This expression pattern is illustrated in the boxplot analysis across adjacent normal, primary TNBC, and lymph node metastatic tissue groups (Figure 1B).

#### 2.1.2. Transcriptome-Guided Prioritization of Candidate miR-548f-3p Downstream Effectors

To identify expression-compatible candidate downstream effectors of miR-548f-3p, differential mRNA expression analyses were performed across three GEO datasets, including GSE45498, GSE76250, and GSE76275. Because miR-548f-3p was downregulated in TNBC, genes showing increased expression in TNBC were prioritized as potential inverse-expression candidates. Differentially expressed genes were identified using an adjusted *p*-value < 0.05 and an absolute log_2_FC ≥ 1.

In the mRNA component of GSE45498, 229 genes were evaluated, among which 95 genes were upregulated and 22 genes were downregulated in primary TNBC tumors compared with adjacent normal tissues. In GSE76250, analysis of 23,912 genes identified 323 differentially expressed genes, including 150 upregulated and 173 downregulated genes in TNBC tumors compared with normal breast tissues. Similarly, in GSE76275, 21,357 genes were assessed, yielding 306 differentially expressed genes, including 123 upregulated and 183 downregulated genes in TNBC compared with non-TNBC breast cancer samples.

Upregulated TNBC-associated genes were then integrated with predicted miR-548f-3p target evidence and regulatory/network-based prioritization. This integrative screening identified complementary candidate sets across the analyzed datasets, including three expression-compatible transcription factor targets, four candidates from the limited GSE45498 mRNA panel, 26 candidates in GSE76275, and 17 candidates in GSE76250. All 17 candidates identified in GSE76250 were also present among the 26 candidates retained from GSE76275, corresponding to a cross-dataset overlap of 65.4%. STRING-based protein–protein interaction analysis subsequently identified a 12-gene interaction network comprising *ME2*, *RPIA*, *PCNA*, *TRIM28*, *ANP32E*, *PLK1*, *CCNA2*, *CKS2*, *SPC25*, *DEPDC1B*, *CDCA7*, and *TFRC* (Figure 1C). This network included genes associated with proliferative, metabolic, chromatin-related, and cell-cycle regulatory processes, supporting their potential relevance to TNBC biology.

Subsequent prioritization using subtype-associated expression patterns identified *ANP32E* and *RPIA* as the top-ranked candidate genes. Both genes showed elevated expression in TNBC compared with normal breast tissue and relatively lower expression in non-TNBC subtypes, supporting a TNBC-enriched expression pattern rather than broad upregulation across all breast cancer subtypes (Figure 1D,E). *RPIA* encodes ribose-5-phosphate isomerase A, a metabolic enzyme involved in the pentose phosphate pathway, whereas *ANP32E* encodes a chromatin-associated H2A.Z regulator with potential relevance to transcriptional plasticity and cell-cycle-associated programs.

**Figure 2:**
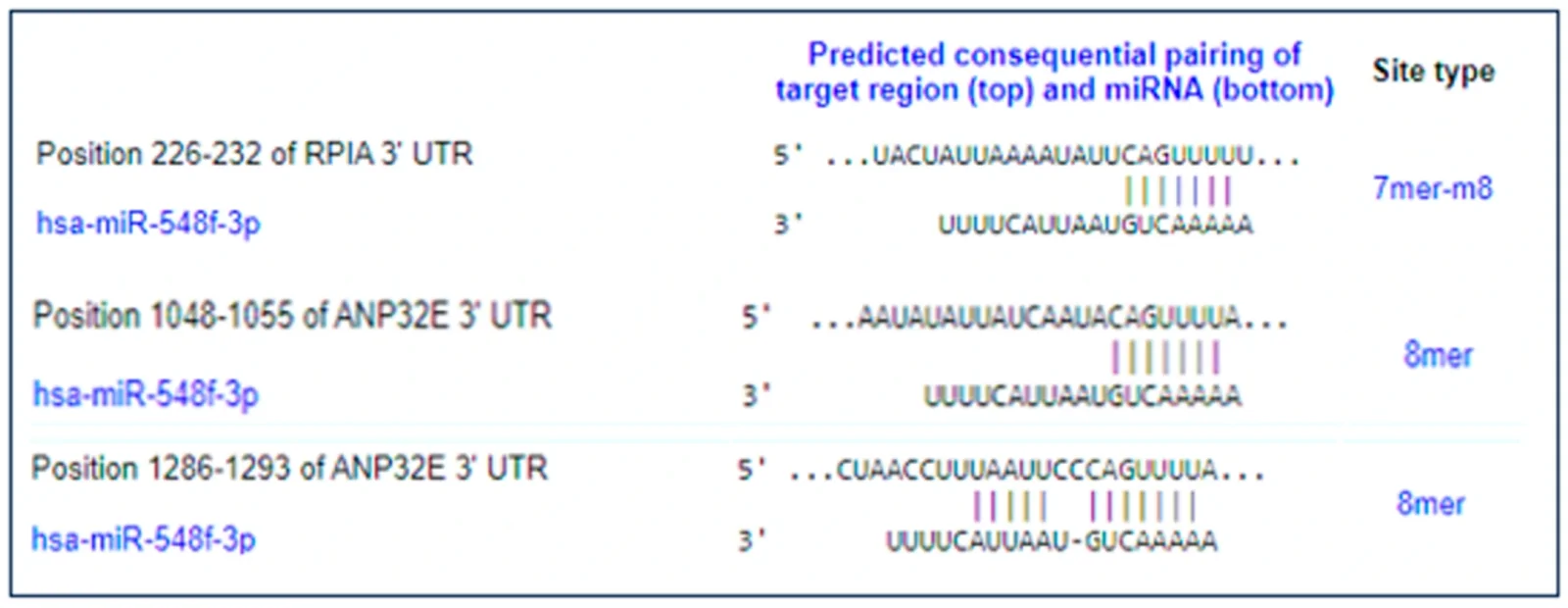
TargetScan-based miRNA target gene prediction analysis.

Among these candidates, *ANP32E* was prioritized for downstream functional evaluation because it showed TNBC-enriched expression, network connectivity with cancer-relevant regulatory genes, predicted miR-548f-3p binding support, and stronger biological relevance to chromatin and cell-cycle regulatory processes. TargetScan-based prediction and structural modeling indicated that *ANP32E* contains two predicted miR-548f-3p binding sites and displays a structurally compatible miRNA–mRNA interaction profile (Figure 2). These findings supported the selection of *ANP32E* as the primary candidate downstream effector for subsequent experimental assessment, with *RPIA* retained as a secondary candidate (Table 2).

#### 2.1.3. In Silico Structural Assessment of Predicted miR-548f-3p Binding Sites Within the ANP32E 3′UTR

In this study, a structure-guided in silico docking framework was applied to evaluate the plausibility of the predicted interaction between miR-548f-3p and its putative downstream target, *ANP32E*. This analysis was informed by the predictive model described by Sweetapple et al. [15], which demonstrated that sequence features, local structural context, seed-pairing architecture, and duplex geometry collectively contribute to miRNA-targeting efficacy. This framework defines structural categories and seed–bulge configurations associated with functional miRNA-mediated repression. Based on these experimentally informed criteria, we applied a comparable structural modeling strategy to the predicted miR-548f-3p/*ANP32E* interaction.

**Figure 3:**
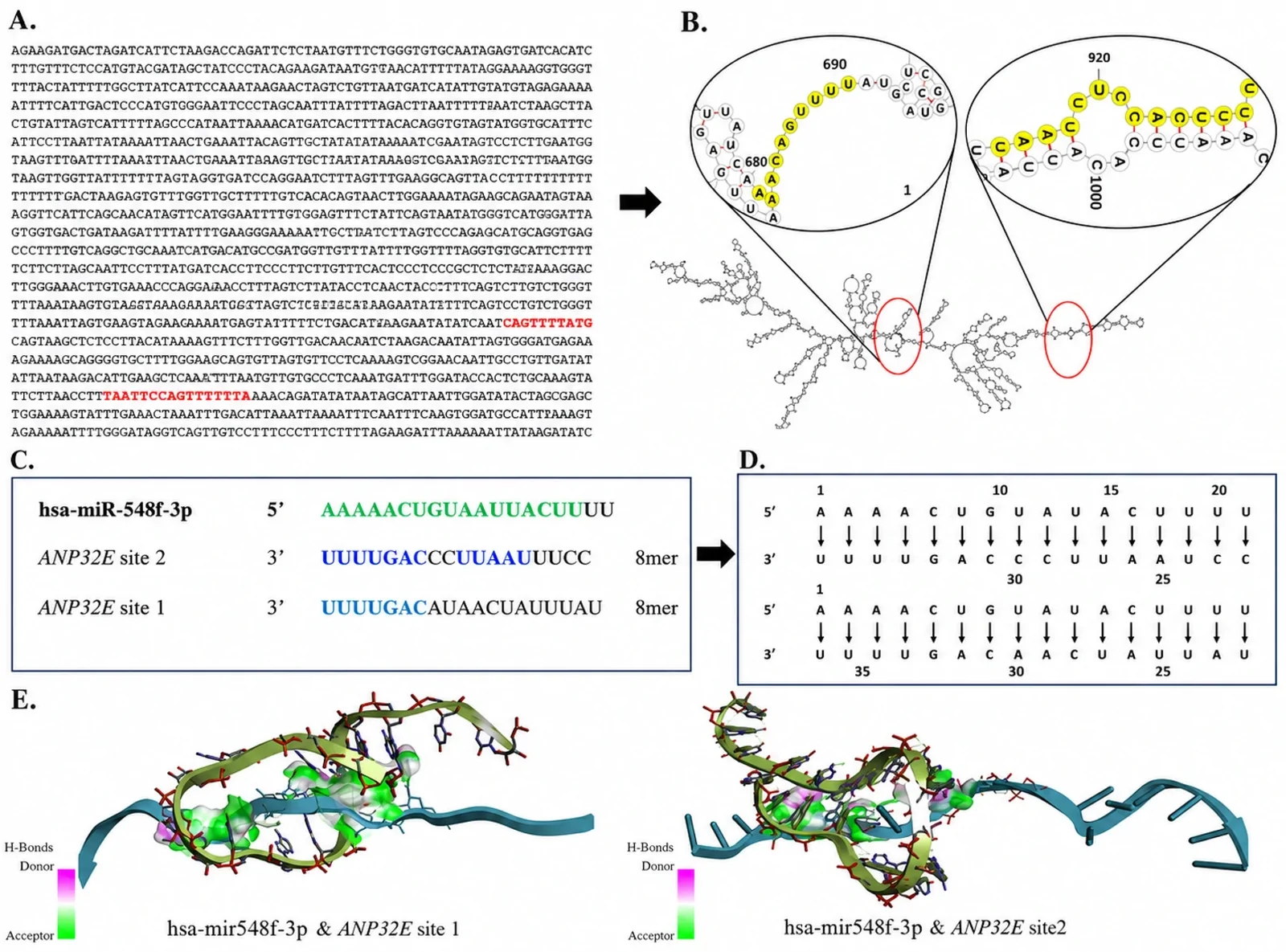
Computational Analysis of miR-548f-3p Interactions with *ANP32E*. (**A**) Transcript structure of *ANP32E* variant 5 showing the exon organization and the 2274 bp 3′UTR containing two predicted miR-548f-3p binding sites. (**B**) MFE-based Vienna analysis indicates both binding sites are accessible. In panel (**B**), yellow highlighting denotes the predicted seed-interaction regions, and red circles indicate bulge/structural features. (**C**) Predicted seed-binding sites within the 3′UTR showing canonical 8-mer seed matches at sites 1 and 2; blue highlighting denotes the predicted seed-match regions. (**D**) RNAcofold and MC-Fold predicted accessible seed regions and conserved bulges (Site 1: ΔG = −28.19 kcal/mol; Site 2: ΔG = −32.2 kcal/mol). (**E**) 3D RNA–RNA docking (HNADOCK) shows geometrically compatible predicted complexes with intermolecular distances < 5 Å and favorable docking scores (Site 1: −206.86; Site 2: −250 ΔG). Visualizations generated in Discovery Studio. Colors in panel (**E**) are used to distinguish structural components in the molecular visualization and do not represent an additional quantitative variable.

Sequence-based and secondary-structure analyses of the predicted miR-548f-3p binding sites within the *ANP32E* transcript showed structural features consistent with miRNA–mRNA duplex geometries associated with functional repression (Figure 3A,B). Minimum free energy (MFE) and local secondary-structure analyses suggested that both predicted binding sites were located within accessible or weakly structured regions of the *ANP32E* mRNA, supporting the thermodynamic plausibility of miR-548f-3p–*ANP32E* duplex formation.

Structurally, site 1 exhibited an extended 8-mer seed match accompanied by a miRNA-associated bulge configuration, whereas site 2 contained a second 8-mer seed match supported by supplementary base pairing beyond the seed region (Figure 3C,D). These features are consistent with structurally favorable miRNA–mRNA interaction patterns described in the Sweetapple classification framework [15]. Separately, descriptive Western blot analysis at 48 h showed lower ANP32E band intensity following miR-548f-3p mimic transfection compared with the NC mimic condition in MCF-7 and MDA-MB-231 cells. Because this protein-level assessment was based on a single biological experiment, it should be interpreted as supportive, semi-quantitative evidence only.

Collectively, the docking-based analyses support the structural plausibility of the predicted *miR-548f-3p*–*ANP32E* interaction and the prioritization of *ANP32E* as a candidate downstream effector. The descriptive protein-level observation is consistent with ANP32E expression responsiveness following miR-548f-3p mimic transfection but does not establish direct target engagement or site-specific 3′UTR-mediated repression. Reporter-based validation will be required to definitively confirm direct 3′UTR-mediated targeting.

#### 2.1.4. ANP32E Is Positively Co-Expressed with E2F/CCNE Cell-Cycle Genes in TNBC

To evaluate whether *ANP32E* expression is associated with proliferative cell-cycle programs in TNBC, Spearman correlation analysis was performed using normalized transcriptomic data from the TNBC subset of GSE76275. *ANP32E* expression showed a significant positive correlation with representative *E2F/CCNE* axis genes, including *E2F1* (Spearman’s ρ = 0.381, FDR = 8.9 × 10^−8^), *CCNE1* (ρ = 0.689, FDR = 1.1 × 10^−28^), and *CCNE2* (ρ = 0.580, FDR = 9.4 × 10^−19^). The strongest association was observed between *ANP32E* and *CCNE1*, suggesting that *ANP32E* expression is linked to G1/S cell-cycle regulatory programs in TNBC. These findings support a positive co-expression relationship between *ANP32E* and *E2F/CCNE*-associated cell-cycle genes (Figure 1F–H).

#### 2.1.5. Single-Cell Analysis Supports ANP32E Enrichment in TNBC-Derived Malignant Epithelial Cells

To determine the cell-type distribution of *ANP32E* expression in breast cancer, single-cell RNA-seq data from the GSE176078 breast cancer atlas were analyzed. Across the dataset, 38,241 cells derived from ER-positive tumors, 19,311 cells derived from HER2-positive tumors, and 42,512 cells derived from TNBC tumors were profiled. Cells were classified into nine major cellular compartments, including malignant epithelial cells, non-malignant epithelial cells, T cells, B cells, myeloid cells, cancer-associated fibroblasts (CAFs), endothelial cells, perivascular cells, and plasmablasts. Cell-type–resolved expression analysis demonstrated that *ANP32E* expression was highest in malignant epithelial cells compared with all other cellular compartments, consistent with a tumor epithelial–dominant expression pattern (Figure 1I).

At the sample level, expression profiles were generated by aggregating normalized single-cell expression within each cell type per TNBC patient. Paired Wilcoxon signed-rank analysis showed a significant increase in *ANP32E* expression in malignant epithelial cells compared with non-malignant epithelial cells (*p* = 0.03, FDR = 0.03), supporting enrichment of *ANP32E* within cancer epithelial compartments (Figure 1J). Within the malignant epithelial compartment, *ANP32E* expression was also significantly higher in cells annotated as Cancer Cycling than in the remaining Cancer Epithelial population from matched TNBC samples (*p* = 0.014, FDR = 0.029), supporting an association between elevated *ANP32E* expression and the cycling malignant-cell phenotype (Figure 1K). These findings suggest that the *ANP32E* enrichment observed in bulk transcriptomic analyses is largely reflected at the single-cell level within malignant epithelial tumor-cell populations and is further associated with the cycling malignant epithelial state, supporting a TNBC-associated tumor-cell expression pattern for *ANP32E*.

### 2.2. Experimental Validation

#### 2.2.1. Demographic and Clinicopathological Characteristics of the Clinical Cohort

The clinical cohort included 60 women with breast cancer, stratified according to TNBC status into triple-negative breast cancer (TNBC, *n* = 30) and non-TNBC breast cancer (*n* = 30) groups. The demographic and clinicopathological characteristics of the patients are summarized in Table 3.

No statistically significant differences were observed between the TNBC and non-TNBC groups in age (*p* = 0.147) or menopausal status (*p* = 0.191). BMI was significantly higher in the TNBC group than in the non-TNBC group (29.5 ± 7.0 vs. 26.2 ± 5.3 kg/m^2^, *p* = 0.047). The distributions of cancer stage, lymph node involvement, and distant metastasis did not differ significantly between the two groups (*p* = 0.307, *p* = 0.795, and *p* = 0.503, respectively). These findings indicate that the two clinical groups were broadly comparable with respect to major clinicopathological characteristics, except for BMI.

#### 2.2.2. Clinical Validation of miR-548f-3p Downregulation and Its Association with ANP32E Expression and Survival Outcome

**Figure 4:**
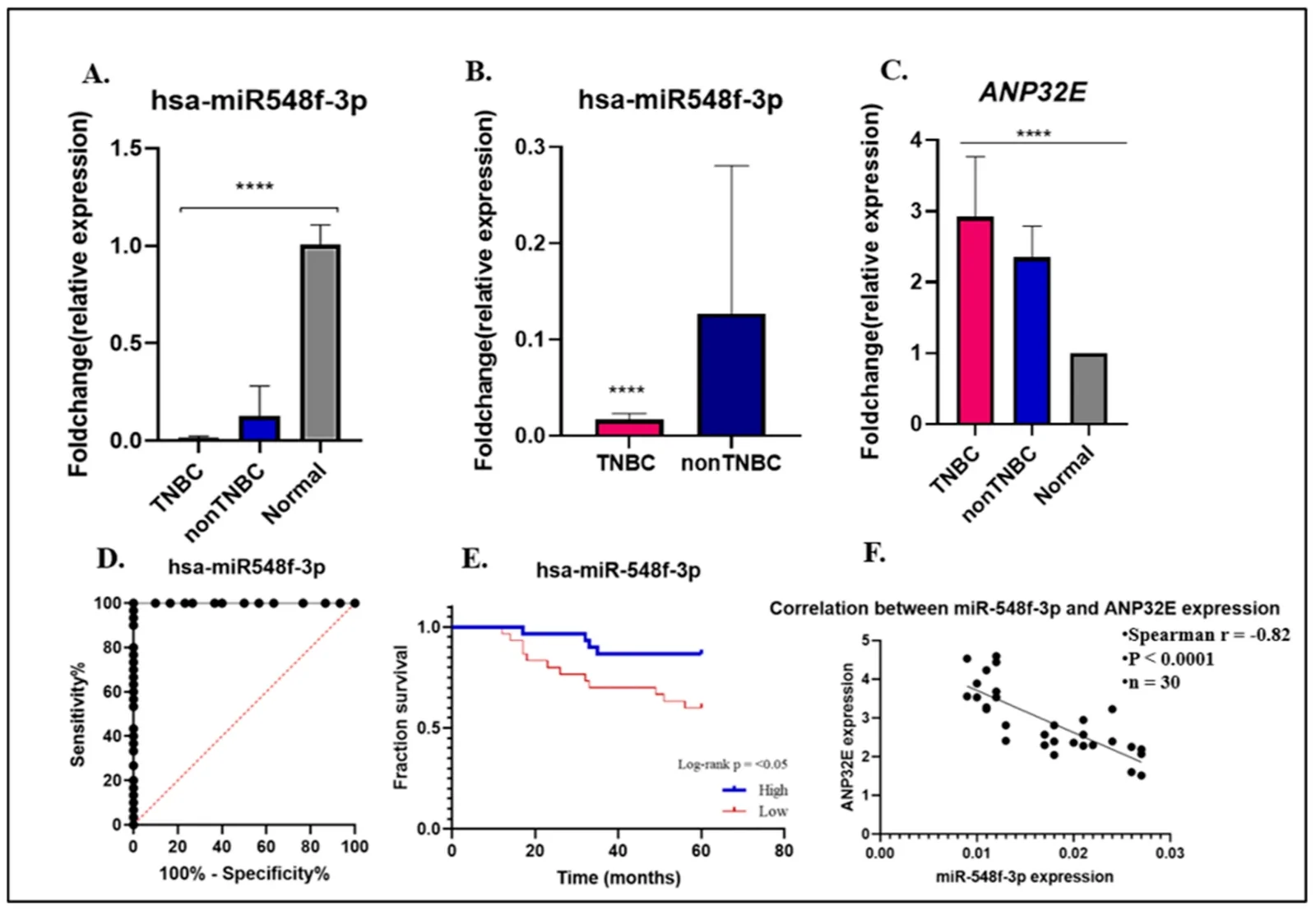
Clinical validation of miR-548f-3p downregulation and its association with *ANP32E* expression, discriminatory performance, and overall survival. (**A**) RT-qPCR analysis of miR-548f-3p expression in TNBC tumors, non-TNBC tumors, and adjacent normal breast tissue controls. Relative expression levels were calculated using the 2^−ΔΔCt^ method. (**B**) Comparison of miR-548f-3p expression between TNBC and non-TNBC tumor samples, showing significantly lower expression in TNBC. (**C**) RT-qPCR analysis of *ANP32E* mRNA expression in TNBC tumors, non-TNBC tumors, and adjacent normal breast tissue controls, showing increased *ANP32E* expression in TNBC tissues. (**D**) Exploratory receiver operating characteristic (ROC) curve analysis of miR-548f-3p expression for discrimination between TNBC and non-TNBC tumor samples within the clinical cohort (AUC = 1.000; stratified-bootstrap 95% CI: 1.000–1.000; 10,000 resamples). (**E**) Kaplan–Meier analysis of 5-year overall survival according to high versus low miR-548f-3p expression using the cohort median as the cutoff (*n* = 60; 30 patients per group). During follow-up, 16 deaths occurred and 44 patients were censored. Survival differences were assessed using the log-rank test (*p* = 0.018). (**F**) Spearman correlation analysis showing an inverse association between miR-548f-3p and *ANP32E* expression in TNBC samples. Each point represents an individual TNBC sample. The clinical cohort comprised 60 patients (30 TNBC and 30 non-TNBC), with patient-matched adjacent normal breast tissues collected from each participant. RT-qPCR measurements were performed in technical triplicate for each tissue sample. Each patient, rather than each technical replicate, was treated as the independent biological unit. Data are shown as mean ± SD where applicable. **** indicates *p* < 0.0001

RT-qPCR analysis was performed using 120 breast tissue specimens obtained from 60 patients, comprising 30 TNBC tumors with 30 patient-matched adjacent normal tissues and 30 non-TNBC tumors with 30 patient-matched adjacent normal tissues. Because no significant difference was observed between the two adjacent normal groups for the analyzed expression markers, the adjacent normal tissues were subsequently considered as a combined reference group for downstream comparisons. Consistent with the downregulation pattern observed in the GSE45498 dataset, miR-548f-3p expression was significantly reduced in TNBC tissues compared with adjacent normal breast tissues (*p* < 0.0001; Figure 4A). miR-548f-3p expression was also significantly lower in TNBC tumors than in non-TNBC tumors (*p* < 0.0001), corresponding to an approximately 7.4-fold decrease in the TNBC group (Figure 4B).

To evaluate whether clinical expression patterns were consistent with the predicted miR-548f-3p–ANP32E candidate relationship, *ANP32E* mRNA expression was assessed by RT-qPCR in the same clinical cohort. *ANP32E* expression was increased in breast tumor tissues, with the highest expression observed in TNBC samples. *ANP32E* expression was significantly higher in TNBC tissues than in adjacent normal controls (*p* < 0.001; Figure 4C). This expression pattern was consistent with an inverse relationship between reduced miR-548f-3p expression and increased *ANP32E* expression in TNBC.

Within the TNBC cohort, the relationship between miR-548f-3p expression and tumor stage was further evaluated. miR-548f-3p expression showed a strong inverse association with increasing tumor stage (Spearman’s ρ = −0.850, *p* < 0.001; *n* = 30), consistent with lower expression in patients with more advanced disease. Consistently, miR-548f-3p expression differed significantly across stages I–IV (Kruskal–Wallis H = 22.04, *p* < 0.001).

Receiver operating characteristic (ROC) curve analysis was performed to evaluate the exploratory within-cohort discriminatory performance of miR-548f-3p expression. miR-548f-3p distinguished TNBC from non-TNBC tumor samples with an AUC of 1.000 (*p* < 0.0001; Figure 4D). The stratified bootstrap 95% confidence interval for the AUC, based on 10,000 resamples, was 1.000–1.000. Because the empirical ROC curve showed complete separation in the present cohort, this degenerate confidence interval should be interpreted cautiously and does not imply perfect diagnostic performance in the broader TNBC population. An optimal cutoff value of 0.036, determined using Youden’s index, yielded apparent sensitivity and specificity of 100% each in the analyzed samples, with exact binomial 95% confidence intervals of 88.43–100.00% for both measures. Repeated stratified five-fold cross-validation with 100 repetitions yielded a mean cross-validated AUC of 1.000, mean sensitivity of 100.0%, mean specificity of 99.9%, and mean accuracy of 99.95%. However, these analyses represent internal assessment within a relatively small cohort of 30 TNBC and 30 non-TNBC tumors. Accordingly, the observed perfect separation should be considered exploratory and potentially cohort-specific rather than evidence of perfect diagnostic performance, and independent validation in larger and more diverse cohorts is required.

Kaplan–Meier survival analysis was performed in a cohort of 60 patients. During the 5-year follow-up, 16 mortality events were observed, while 44 patients were censored. Patients in the higher miR-548f-3p expression group showed significantly better overall survival compared with those in the lower-expression group (log-rank *p* = 0.018; Figure 4E). In an exploratory univariable Cox proportional hazards analysis, the higher miR-548f-3p expression group was associated with a lower observed hazard of death (HR = 0.28, 95% CI: 0.09–0.87, *p* = 0.028). However, model discrimination was modest (C-index = 0.651), and the limited number of mortality events substantially constrained robust multivariable assessment of independent prognostic value.

As a limited sensitivity analysis, a parsimonious Cox model including BMI as a continuous covariate was also examined. The association remained similar in magnitude after adjustment for BMI (adjusted HR = 0.31, 95% CI: 0.10–0.99, *p* = 0.048), whereas BMI itself was not significantly associated with overall survival (HR = 1.04 per kg/m^2^, 95% CI: 0.96–1.12, *p* = 0.340). In a separate parsimonious model including age as a continuous covariate, the association was similarly retained (adjusted HR = 0.264, 95% CI: 0.085–0.822, *p* = 0.022), while age was not significantly associated with overall survival (HR = 1.022 per year, 95% CI: 0.981–1.064, *p* = 0.297). Finally, in an additional model simultaneously including age and BMI, the effect estimate for higher miR-548f-3p expression remained comparable (adjusted HR = 0.292, 95% CI: 0.091–0.933, *p* = 0.038); neither age (HR = 1.020 per year, 95% CI: 0.979–1.063, *p* = 0.349) nor BMI (HR = 1.034 per kg/m^2^, 95% CI: 0.956–1.117, *p* = 0.404) was significantly associated with overall survival. These sensitivity analyses were not intended to establish independent prognostic value, and residual confounding by tumor stage, treatment, and other clinicopathological factors cannot be excluded. For the BMI-adjusted Cox model, the proportional hazards assumption was assessed using Schoenfeld residuals, with no evidence of violation (global *p* = 0.680).

Correlation analysis further demonstrated a strong inverse association between miR-548f-3p and *ANP32E* expression in TNBC samples. Spearman’s rank correlation analysis showed a markedly negative correlation between miR-548f-3p and *ANP32E* expression levels (ρ = −0.8245, 95% CI: −0.9153 to −0.6541, *p* < 0.0001; *n* = 30; Figure 4F). This inverse expression relationship supports the hypothesis that *ANP32E* may represent a downstream effector associated with miR-548f-3p loss in TNBC.

#### 2.2.3. miR-548f-3p Mimic Restoration Reduces ANP32E mRNA Expression in Breast Cancer Cell Lines

**Figure 5:**
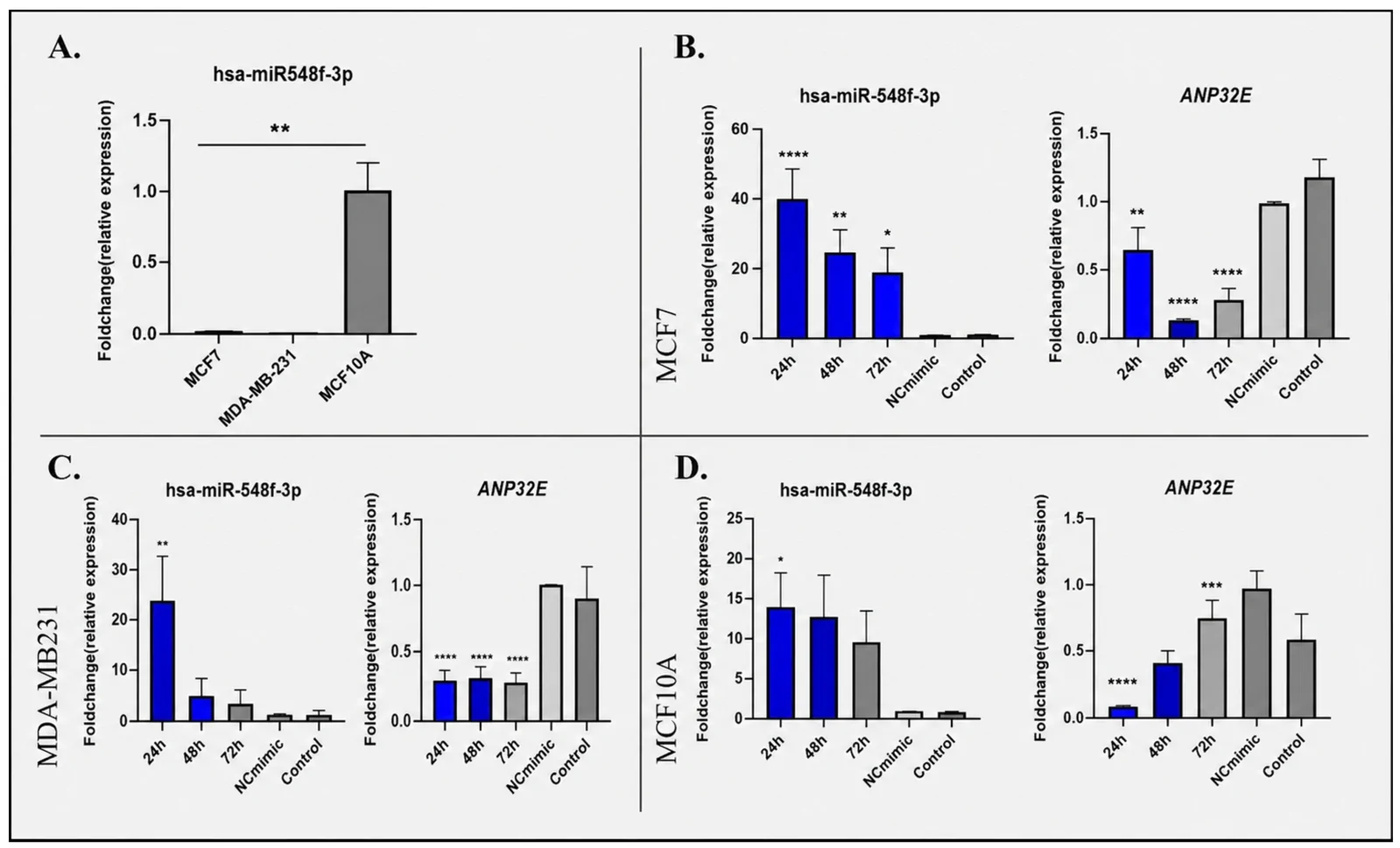
RT-qPCR analysis of miR-548f-3p restoration and *ANP32E* mRNA modulation in breast cell lines. (**A**) Basal expression of miR-548f-3p in MCF-7, MDA-MB-231, and MCF-10A cells. miR-548f-3p expression was lower in tumor-derived breast cancer cell lines than in non-tumorigenic MCF-10A cells. (**B**–**D**) Time-course RT-qPCR analysis following miR-548f-3p mimic transfection in MCF-7 (**B**), MDA-MB-231 (**C**), and MCF-10A (**D**) cells. Left panels show miR-548f-3p expression at 24, 48, and 72 h post-transfection, confirming efficient mimic-mediated restoration. Right panels show *ANP32E* mRNA expression at the corresponding time points. Relative expression levels were calculated using the 2^−ΔΔCt^ method. Data are presented as mean ± SD from three independent biological replicates, each measured in technical triplicate. For the time-course experiments, statistical comparisons were performed using ordinary one-way ANOVA followed by Dunnett’s multiple-comparisons test, with the NC mimic group used as the reference control. Reported *p* values were adjusted for multiple comparisons. ns, not significant; * *p *< 0.05, ** *p *< 0.01, *** *p* < 0.001; **** *p* < 0.0001.

Baseline RT-qPCR analysis showed that endogenous miR-548f-3p expression was markedly lower in the breast cancer cell lines MDA-MB-231 and MCF-7 than in the non-tumorigenic mammary epithelial cell line MCF-10A (overall *p* = 0.0036; Figure 5A). This expression pattern supported the use of mimic-based restoration to investigate the potential regulatory effect of miR-548f-3p in tumor-derived breast cancer cell models.

Transient transfection with a synthetic miR-548f-3p mimic induced a strong increase in miR-548f-3p expression across all three cell lines. In MCF-7, MDA-MB-231, and MCF-10A cells, miR-548f-3p expression reached its highest level at 24 h post-transfection and then gradually declined at 48 and 72 h, consistent with the transient expression kinetics of synthetic miRNA mimics (Figure 5B–D, left panels). In MCF-7 cells, miR-548f-3p expression was significantly increased relative to the NC mimic group at 24, 48, and 72 h post-transfection (*p* < 0.0001, *p* = 0.0023, and *p* = 0.0108, respectively; Figure 5B, left panel). In MDA-MB-231 cells, a significant increase in miR-548f-3p expression relative to the NC mimic group was observed at 24 h post-transfection (*p* = 0.0040), whereas the increases at 48 and 72 h did not reach statistical significance (Figure 5C, left panel). In MCF-10A cells, miR-548f-3p expression was significantly increased at 24 h post-transfection relative to the NC mimic group (*p* = 0.0374), whereas the changes observed at 48 and 72 h were not statistically significant (Figure 5D, left panel).

In tumor-derived breast cancer cell lines, miR-548f-3p mimic transfection was accompanied by reduced *ANP32E* mRNA expression. In MCF-7 cells, *ANP32E* expression decreased following miR-548f-3p mimic transfection, with the most pronounced reduction observed at 48 h post-transfection and significant reductions detected at 24, 48, and 72 h relative to the NC mimic group (*p* = 0.0013, *p* < 0.0001, and *p* < 0.0001, respectively; Figure 5B, right panel). In MDA-MB-231 cells, *ANP32E* expression was consistently reduced across the 24, 48, and 72 h time points relative to the NC mimic group, with significant reductions observed at all three time points (*p* < 0.0001 for all comparisons; Figure 5C, right panel). These findings indicate that restoration of miR-548f-3p is associated with suppression of *ANP32E* mRNA expression in breast cancer cell lines.

In MCF-10A cells, miR-548f-3p mimic transfection also altered *ANP32E* expression; however, the response was more variable over time than that observed in tumor-derived breast cancer cells. *ANP32E* downregulation was most evident at 24 h and 72 h post-transfection, whereas the reduction was less pronounced at 48 h. Relative to the NC mimic group, *ANP32E* expression was significantly reduced at 24 h and 72 h (*p* < 0.0001 and *p* = 0.0008, respectively), whereas the change at 48 h was not statistically significant (Figure 5D, right panel). Overall, these data suggest that miR-548f-3p-mediated modulation of *ANP32E* is more consistent in malignant breast cancer cell lines than in non-tumorigenic mammary epithelial cells.

Collectively, these results confirm efficient miR-548f-3p mimic transfection and show that miR-548f-3p restoration is associated with reduced *ANP32E* mRNA expression, supporting *ANP32E* as an expression-responsive candidate downstream effector of miR-548f-3p in breast cancer cells.

#### 2.2.4. Mimic-Mediated Restoration of miR-548f-3p Promotes Apoptosis and Induces G_0_/G_1_ Accumulation

**Figure 6:**
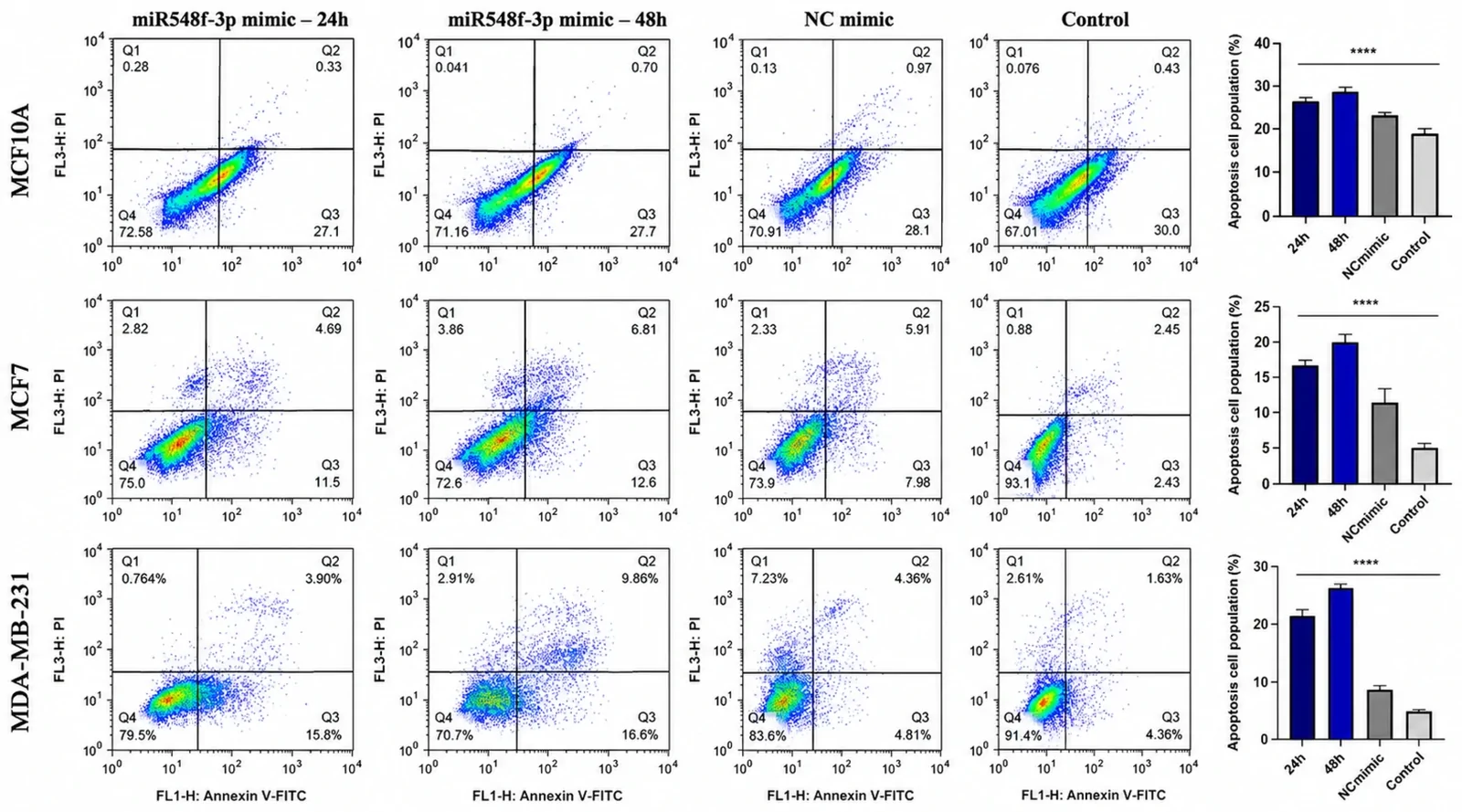
miR-548f-3p restoration promotes apoptosis in breast cell lines. Annexin V–FITC/propidium iodide (PI) flow-cytometry analysis was performed in MCF-10A, MCF-7, and MDA-MB-231 cells following transfection with miR-548f-3p mimic for 24 and 48 h. Untreated cells and NC mimic-transfected cells were included as controls. Representative dot plots show viable cells (Annexin V^−^/PI^−^), early apoptotic cells (Annexin V^+^/PI^−^), late apoptotic/secondary necrotic cells (Annexin V^+^/PI^+^), and necrotic cells (Annexin V^−^/PI^+^). Bar graphs show the quantification of total apoptotic cells, calculated as the sum of early and late apoptotic populations. Data are presented as mean ± SD from three independent biological replicates. Statistical significance was assessed using the Kruskal–Wallis test (*p* < 0.0001), followed by post hoc multiple-comparisons testing; significant pairwise comparisons are indicated in the corresponding panels. **** indicates *p* < 0.0001. The pseudocolor gradient in the representative flow-cytometry plots represents relative event density.

To determine whether miR-548f-3p restoration affects cell survival, apoptosis was assessed by Annexin V–FITC/PI flow cytometry at 24 and 48 h after mimic transfection. miR-548f-3p mimic transfection increased the total apoptotic fraction, defined as early plus late apoptotic cells, in MDA-MB-231, MCF-7, and MCF-10A cells compared with untreated and NC mimic-transfected controls (Figure 6). The apoptotic response was most evident at 48 h post-transfection and was particularly pronounced in the tumor-derived breast cancer cell lines MDA-MB-231 and MCF-7. An increase in apoptosis was also observed in non-tumorigenic MCF-10A cells, although the magnitude and baseline apoptotic profile differed from those observed in malignant breast cancer cells.

**Figure 7:**
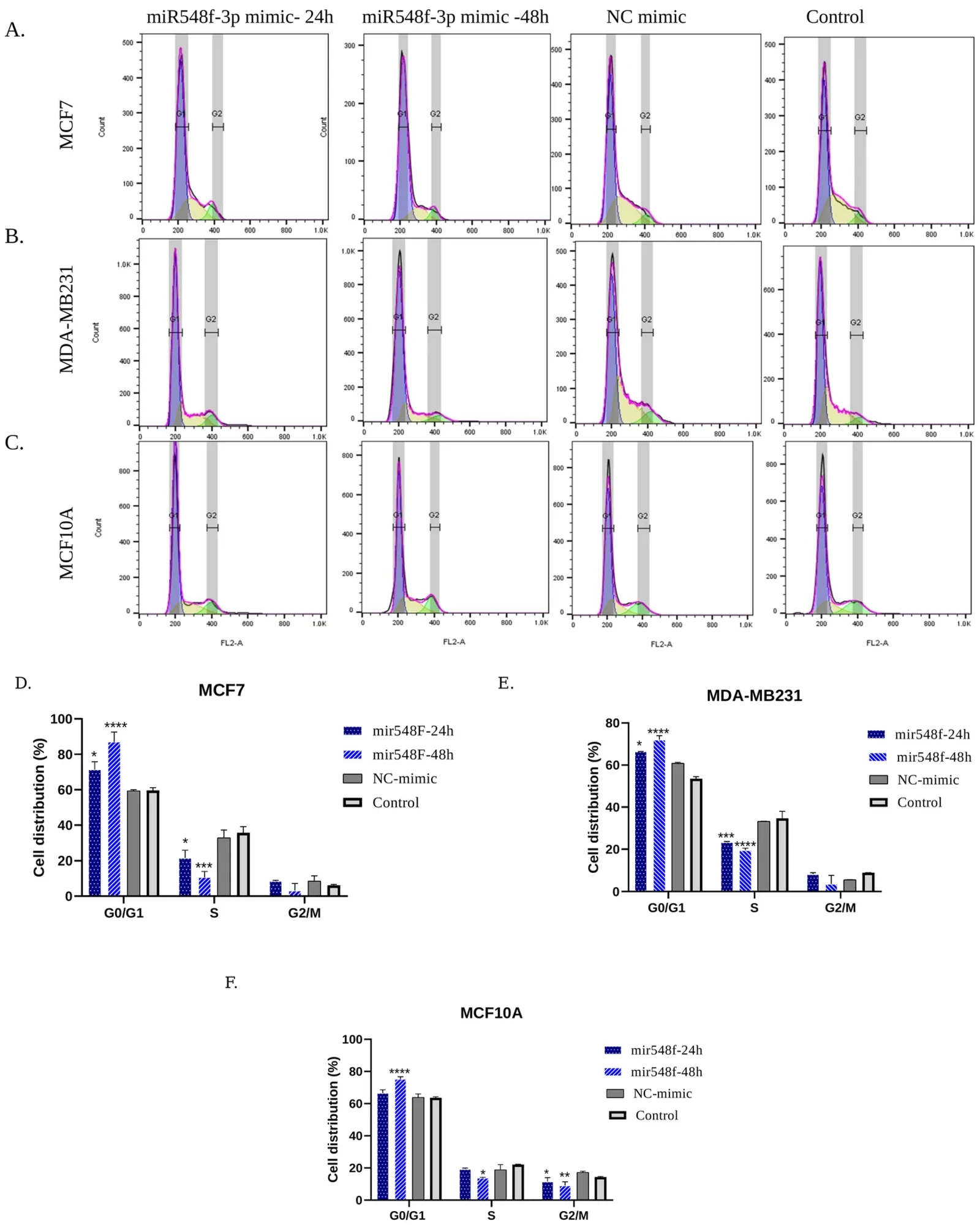
miR-548f-3p restoration alters cell-cycle distribution in breast cancer cells. Cell-cycle distribution was assessed by propidium iodide-based DNA-content flow cytometry in MCF-7, MDA-MB-231, and MCF-10A cells 24 and 48 h after transfection with miR-548f-3p mimic. Untreated and NC mimic-transfected cells served as controls. (**A**–**C**) Representative DNA-content histograms for MCF-7, MDA-MB-231, and MCF-10A cells, respectively. (**D**–**F**) Corresponding quantification of G0/G1, S, and G2/M populations for MCF-7, MDA-MB-231, and MCF-10A cells, respectively. In the quantitative bar graphs, dark blue indicates miR-548f-3p mimic at 24 h, blue hatched bars indicate miR-548f-3p mimic at 48 h, gray indicates NC mimic, and white outlined bars indicate untreated control. The colored overlays in the representative DNA-content histograms indicate the fitted cell-cycle phase distributions and do not represent additional treatment groups. In MCF-7 cells, significant differences versus NC mimic were detected in G_0_/G_1_ at 24 h (*p* = 0.0119) and 48 h (*p* < 0.0001), and in S phase at 24 h (*p* = 0.0171) and 48 h (*p* = 0.0001). In MDA-MB-231 cells, significant differences were observed in G_0_/G_1_ at 24 h (*p* = 0.0175) and 48 h (*p* < 0.0001), and between NC mimic and control (*p* = 0.0026), as well as in S phase at 24 h (*p* = 0.0002) and 48 h (*p* < 0.0001). In MCF-10A cells, significant differences were observed in G_0_/G_1_ at 48 h (*p* < 0.0001), S phase at 48 h (*p* = 0.0260), and G_2_/M at 24 h (*p* = 0.0137) and 48 h (*p* = 0.0012). Data are presented as mean ± SD from three independent experiments. Statistical significance was determined relative to the NC mimic group unless otherwise indicated. * *p* < 0.05; ** *p* < 0.01; *** *p* < 0.001; **** *p* < 0.0001.

Given the association of *ANP32E* with cell-cycle regulatory programs, we next evaluated whether miR-548f-3p restoration altered cell-cycle distribution. Flow-cytometric DNA-content analysis showed that miR-548f-3p mimic transfection increased the proportion of cells in the G_0_/G_1_ phase, accompanied by a reduction in the S-phase fraction, particularly in MCF-7 and MDA-MB-231 cells (Figure 7). This pattern was most prominent at 48 h post-transfection and is consistent with impaired G_1_/S cell-cycle progression. MCF-10A cells also showed a modest shift toward G_0_/G_1_ accumulation, although the effect was less pronounced than in tumor-derived breast cancer cells.

Together, these findings indicate that miR-548f-3p restoration promotes apoptotic cell death and disrupts cell-cycle progression in breast cancer cell models, supporting a tumor-suppressive functional role for miR-548f-3p.

#### 2.2.5. miR-548f-3p Restoration Suppresses Migration-Associated Wound Closure in Breast Cancer Cells

**Figure 8:**
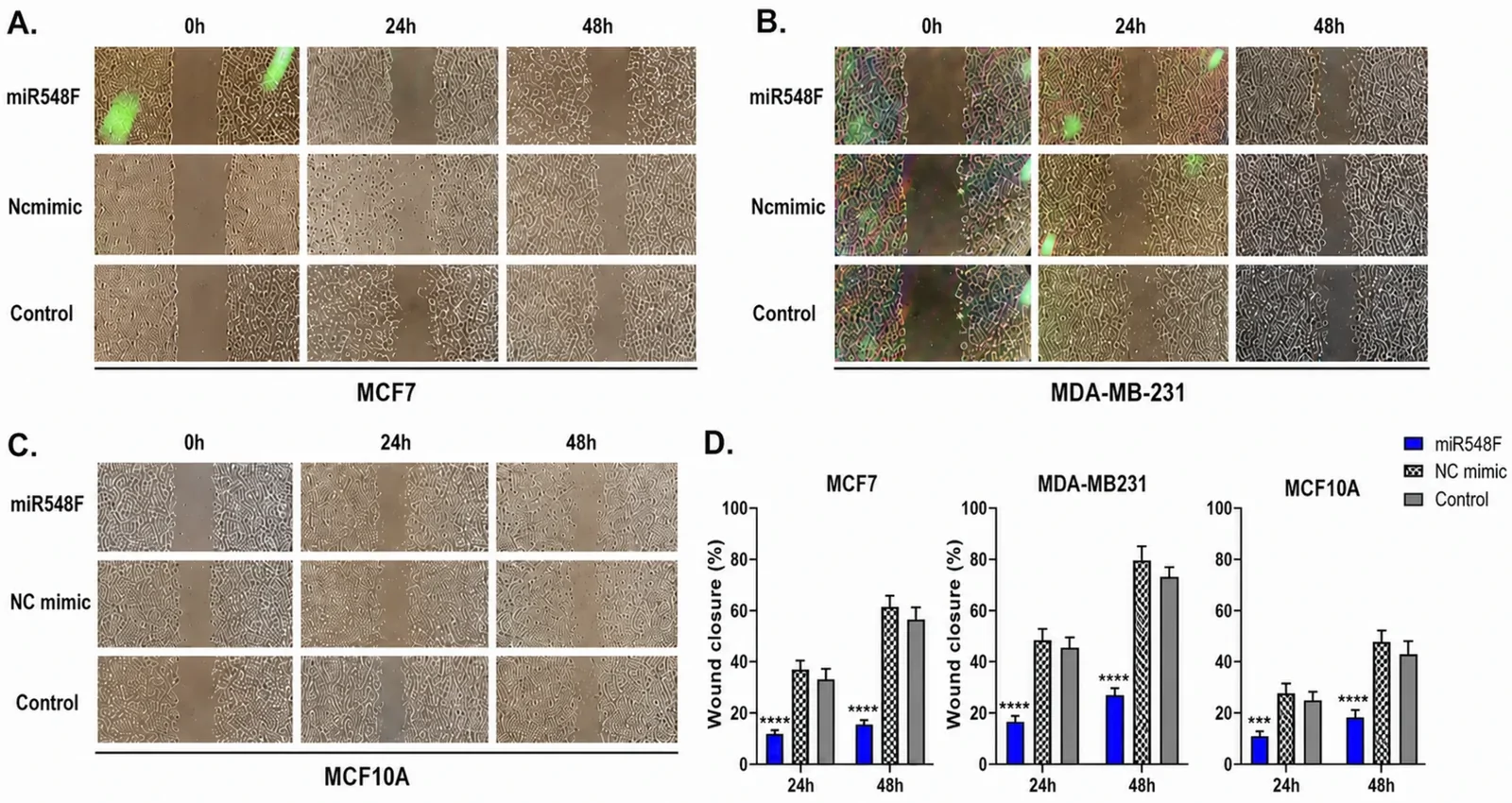
miR-548f-3p restoration suppresses migration-associated wound closure in breast cell lines. Representative wound-healing images of MCF-7 (**A**), MDA-MB-231 (**B**), and non-tumorigenic MCF-10A (**C**) cells captured at 0, 24, and 48 h after scratch generation following transfection with miR-548f-3p mimic or negative control mimic. Untreated cells were included as controls. Images were acquired at 10× magnification. (**D**) Quantification of wound closure percentage at 24 and 48 h. Relative to the NC mimic group, miR-548f-3p restoration significantly reduced wound closure in MCF-7 cells at both 24 and 48 h (*p* < 0.0001), in MDA-MB-231 cells at both 24 and 48 h (*p* < 0.0001), and in MCF-10A cells at 24 h (*p* = 0.0008) and 48 h (*p* < 0.0001). Data are presented as mean ± SD from three independent experiments. Statistical comparisons were performed using two-way ANOVA followed by appropriate multiple-comparison testing. *** *p *< 0.001, **** *p* < 0.0001.

Wound-healing assays were performed to evaluate the effect of miR-548f-3p restoration on migration-associated wound closure in breast cell lines. In MCF-7 cells, miR-548f-3p mimic transfection significantly reduced wound closure at both 24 and 48 h after scratch generation compared with NC mimic-transfected and untreated control cells (Figure 8A,D). A comparable inhibitory effect was observed in the TNBC cell line MDA-MB-231, in which miR-548f-3p mimic-transfected cells showed markedly delayed wound closure and a wider residual scratch area at both time points relative to control conditions (Figure 8B,D).

In the non-tumorigenic MCF-10A cell line, miR-548f-3p mimic transfection also reduced wound closure at 24 and 48 h, although the overall wound-closure dynamics differed from those observed in tumor-derived breast cancer cells (Figure 8C,D). These findings indicate that miR-548f-3p restoration impairs wound closure in breast cancer cell models, particularly in MDA-MB-231 cells, indicating reduced migration-associated wound closure under the experimental conditions used. Because wound closure can be influenced by both cell migration and proliferative capacity, these results were interpreted together with the apoptosis and cell-cycle findings.

#### 2.2.6. miR-548f-3p Restoration Is Associated with Lower Transwell Migration- and Invasion-Associated Cell Counts in Breast Cancer Cells

**Figure 9:**
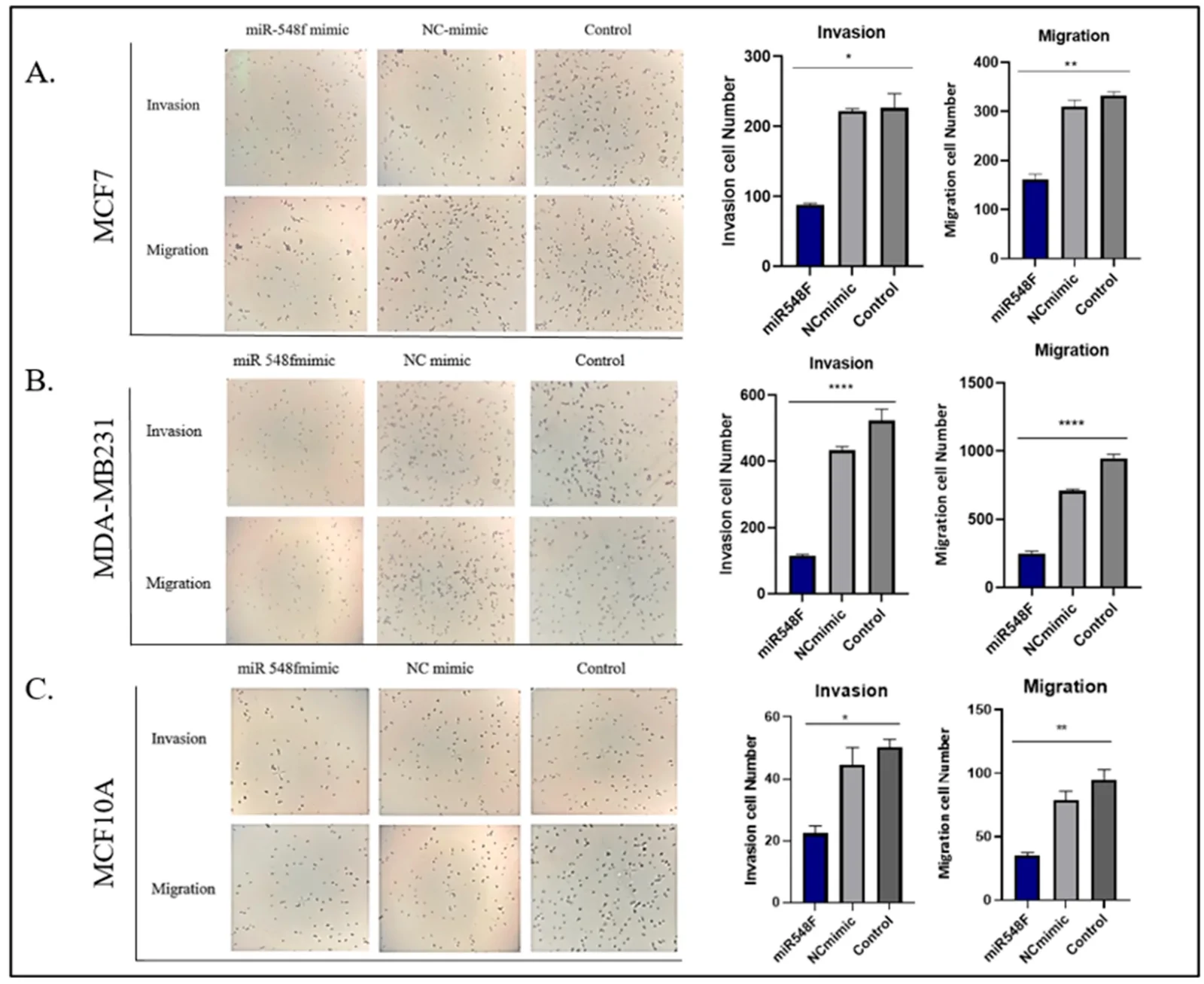
miR-548f-3p restoration is associated with lower Transwell migration- and invasion-associated cell counts in breast cell lines. Representative images and quantification of Transwell invasion and migration assays in MCF-7 (**A**), MDA-MB-231 (**B**), and non-tumorigenic MCF-10A (**C**) cells following transfection with miR-548f-3p mimic or negative control mimic. Untreated cells were included as controls. For invasion assays, inserts were coated with Matrigel, whereas uncoated inserts were used for migration assays. Complete medium containing 10% FBS in the lower chamber was used as a chemoattractant. Migrated and invaded cells were fixed, stained with crystal violet, and quantified by counting five randomly selected microscopic fields per insert. Omnibus Kruskal–Wallis tests showed overall differences among the treatment groups for migration and invasion in MCF-7 cells (*p* = 0.0071 and *p* = 0.0490, respectively), MDA-MB-231 cells (*p* < 0.0001 for both assays), and MCF-10A cells (*p* = 0.0036 and *p* = 0.0143, respectively). These *p* values represent omnibus tests and do not establish specific pairwise differences between treatment groups. Data are presented as mean ± SD from three independent biological replicates. * *p* < 0.05; ** *p* < 0.01; **** *p* < 0.0001.

Transwell migration and Matrigel-coated Transwell invasion assays were performed to further evaluate migration- and invasion-associated phenotypes following miR-548f-3p restoration. In MCF-7 cells, the omnibus Kruskal–Wallis test indicated overall differences among the treatment groups for both migration (*p* = 0.0071) and invasion (*p* = 0.0490). The miR-548f-3p mimic group showed lower migrated and invaded cell counts than the NC mimic and untreated groups (Figure 9A); however, these omnibus tests do not by themselves establish the significance of specific pairwise group differences.

In MDA-MB-231 cells, significant overall group differences were likewise observed for both migration and invasion (Kruskal–Wallis *p* < 0.0001 for each assay). The lowest migrated and invaded cell counts were observed in the miR-548f-3p mimic group (Figure 9B).

In non-tumorigenic MCF-10A cells, overall group differences were also detected for migration (*p* = 0.0036) and invasion (*p* = 0.0143), with lower cell counts observed in the miR-548f-3p mimic group (Figure 9C). Collectively, these findings show treatment-associated differences in Transwell migration- and invasion-associated cell counts, with the miR-548f-3p mimic condition displaying the lowest counts across the examined cell models. Because the reported Kruskal–Wallis *p* values represent omnibus tests, the present analysis should not be interpreted as establishing specific pairwise differences unless supported by post hoc comparisons. In addition, given the pro-apoptotic and cell-cycle effects observed following miR-548f-3p restoration, the reduced Transwell cell counts may reflect combined effects on migratory/invasive behavior and overall cellular fitness.

#### 2.2.7. Descriptive Assessment of Reduced ANP32E Protein Expression Following miR-548f-3p Restoration in Breast Cancer Cells

**Figure 10:**
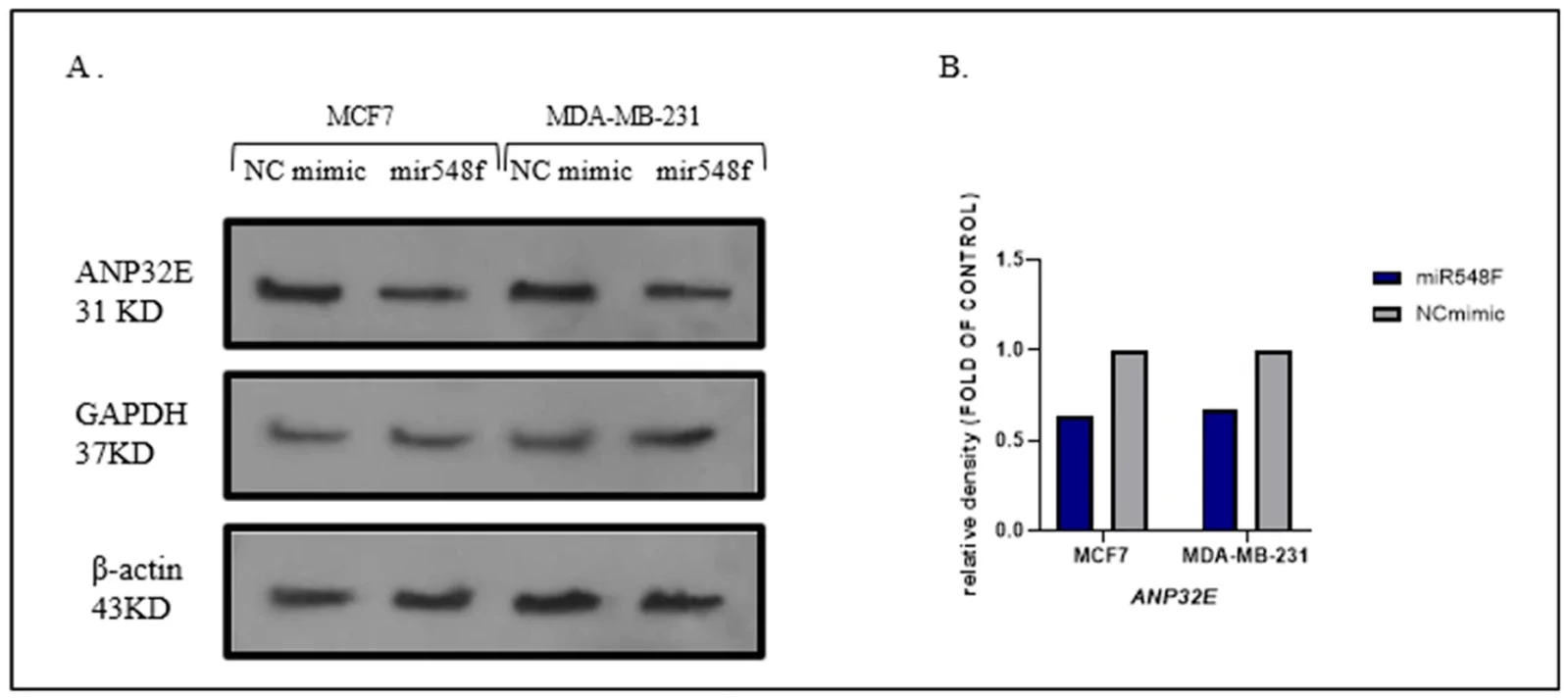
Descriptive assessment of ANP32E protein abundance following miR-548f-3p mimic transfection. (**A**) Western blot analysis of ANP32E in MCF-7 and MDA-MB-231 cells at 48 h after transfection with miR-548f-3p mimic or negative-control mimic. Full-length Western blot images corresponding to the cropped panels shown here are provided in Supplementary Figure S1. GAPDH and β-actin were assessed as loading controls; β-actin was used for normalization. (**B**) Descriptive densitometric analysis of ANP32E band intensity normalized to β-actin and expressed relative to the NC mimic condition. The Western blot represents a single biological experiment; therefore, the densitometric values are presented descriptively and no inferential statistical testing or *p* values were applied.

Western blotting was performed at a single post-transfection endpoint (48 h) to descriptively assess ANP32E protein abundance following miR-548f-3p mimic transfection. In the representative experiment, lower ANP32E band intensity was observed in miR-548f-3p mimic-transfected cells compared with NC mimic-transfected cells in both MCF-7 and MDA-MB-231 cells (Figure 10A). Descriptive densitometric analysis normalized to β-actin showed the same directional reduction in relative ANP32E band intensity (Figure 10B). Because the Western blot analysis was based on a single biological experiment, these densitometric values were not subjected to inferential statistical testing and should be interpreted as semi-quantitative descriptive observations rather than as statistically validated protein-level effects. β-actin was used for normalization because the apparent molecular mass of GAPDH was relatively close to that of ANP32E under the electrophoretic conditions used. These observations are consistent with the direction of the ANP32E mRNA response following miR-548f-3p mimic transfection; however, additional independent biological replicates and protein time-course analyses will be required for quantitative confirmation of the protein-level response.

## 3. Discussion

Triple-negative breast cancer (TNBC) remains a major therapeutic challenge due to its marked molecular heterogeneity, aggressive clinical behavior, early therapeutic resistance, and lack of broadly actionable ER-, PR-, and HER2-directed targets [16]. These features have shifted increasing attention toward regulatory genomic and epigenomic mechanisms that sustain transcriptional plasticity, lineage adaptability, and malignant progression. In this context, microRNAs (miRNAs) have emerged as attractive regulatory and therapeutic candidates because of their ability to coordinately modulate multi-gene oncogenic programs at the post-transcriptional level without directly altering the underlying genomic sequence [17].

Within this regulatory framework, the miR-548 family represents one of the largest and evolutionarily diverse primate-specific miRNA families in the human genome [13]. Members of this family are widely dispersed across human chromosomes, a genomic distribution consistent with their proposed derivation and expansion from MADE1 miniature inverted-repeat transposable elements (MITEs) [13]. This transposable-element-associated origin may have contributed to both the extensive genomic distribution and sequence diversification of the miR-548 family. Importantly, although substantial sequence homology among family members may result in partially overlapping target repertoires and potential functional redundancy, such redundancy is unlikely to be uniform across the family. Variation in the 5′ends of mature miR-548 sequences and associated seed-shifting events can alter seed composition and thereby diversify target recognition among individual family members [13]. Thus, related miR-548 miRNAs may share subsets of regulatory targets while retaining distinct target specificities.

Previous studies have reported tumor-suppressive roles for several miR-548 family members, including miR-548b and miR-548d, across breast cancer contexts [13,18,19], highlighting the broader regulatory relevance of this family. Based on these observations, the miR-548 family was selected a priori for focused investigation. In our family-focused analysis, seven miR-548 members showed FDR-significant downregulation in TNBC compared with adjacent normal tissues. Among these candidates, miR-548f-3p was prioritized because of its FDR-supported downregulation and comparatively limited characterization in breast cancer, particularly TNBC, and its reduced expression was subsequently confirmed in the clinical cohort.

The present study did not systematically compare the target repertoires or functional activities of the other downregulated miR-548 members. Consequently, the extent to which these miRNAs exhibit functional redundancy, cooperative regulation, or competition for overlapping target interactions cannot be determined from the current data. Although shared regulation of *ANP32E* by other miR-548 family members has not been established in breast cancer, their sequence relatedness raises the possibility of partially overlapping regulatory networks. Conversely, seed-sequence variation among family members may confer distinct target specificities. Comparative target analysis and combinatorial perturbation of multiple miR-548 family members will therefore be required to determine whether their regulatory effects in TNBC are redundant, cooperative, competitive, or functionally distinct. Our findings extend these observations by implicating miR-548f-3p as a miRNA with potential tumor-suppressive activity in TNBC, thereby contributing to the emerging landscape of miRNA-mediated post-transcriptional regulation in aggressive breast cancer. This conclusion was supported by integrative bioinformatic analyses and validation in patient-derived tissue samples, both of which demonstrated reduced miR-548f-3p expression in TNBC contexts. In contrast, *ANP32E*, prioritized as a candidate downstream effector of miR-548f-3p, showed increased expression in TNBC and displayed a significant inverse correlation with miR-548f-3p expression in TNBC patient samples. This inverse expression pattern supports the biological plausibility of a miR-548f-3p/*ANP32E* regulatory relationship, while not by itself establishing direct 3′UTR-mediated targeting. In addition, miR-548f-3p expression showed a significant inverse association with tumor stage within the TNBC cohort, consistent with lower expression in more advanced disease. Given the limited cohort size, however, this clinicopathological association should be considered exploratory and requires confirmation in larger cohorts.

ANP32E has been described in prior studies as an H2A.Z-specific histone chaperone involved in H2A.Z eviction, chromatin accessibility, E2F-associated transcriptional programs, and ATR-mediated replication stress signaling—processes that may contribute to proliferative behavior and tumor adaptability in TNBC [14,20,21]. These mechanistic functions, however, were established in independent studies and were not directly interrogated in the present work. In our analysis, *ANP32E* expression showed a consistent positive association with *E2F/CCNE* cell-cycle regulatory genes, including *E2F1*, *CCNE1*, and *CCNE2*, in TNBC transcriptomic data. In parallel, single-cell RNA-seq analysis of the GSE176078 breast cancer atlas showed that *ANP32E* expression was enriched predominantly in malignant epithelial cell populations. Within the malignant epithelial compartment, *ANP32E* expression was also higher in cells annotated as Cancer Cycling than in the remaining Cancer Epithelial population, supporting an association with the cycling malignant-cell phenotype. Collectively, these findings support a tumor-cell-enriched and proliferation-associated expression pattern of *ANP32E* in TNBC. From a broader genetic perspective, the present findings should be distinguished from, but interpreted alongside, previous genome-wide association studies of TNBC susceptibility. Earlier GWAS and large-scale genetic association studies identified multiple germline susceptibility loci associated with TNBC risk, including loci involving *ESR1*, *TOX3*, *RAD51L1*, 19p13.1, *PTHLH*, *TERT*, and *MDM4*, supporting a distinct and polygenic inherited susceptibility architecture for this breast cancer subtype [22,23]. More recent subtype-specific GWAS have further demonstrated substantial heterogeneity in genetic susceptibility across breast cancer subtypes [24]. The present study does not directly replicate these germline associations because it addresses a different regulatory layer, namely tumor-associated miRNA and gene expression dysregulation. Neither miR-548f-3p nor ANP32E has been established as a TNBC-specific lead susceptibility locus in these representative GWAS; therefore, direct locus-level concordance should not be inferred. However, an eQTL- and GWAS-based Mendelian randomization study identified ANP32E among genes whose genetically predicted expression was associated with overall breast cancer risk [25]. Importantly, reverse Mendelian randomization in that study provided evidence consistent with reverse causation involving ANP32E, and the association was not TNBC-specific. Accordingly, these genetic data should be regarded as complementary but non-conclusive context for ANP32E rather than evidence that it represents an inherited TNBC susceptibility gene. Taken together, previous GWAS and the present findings highlight distinct but potentially complementary layers of TNBC biology, encompassing inherited genetic susceptibility and tumor-associated post-transcriptional regulation. Functionally, mimic-mediated elevation of miR-548f-3p altered multiple cancer-associated phenotypic readouts, including apoptosis, cell-cycle progression, migration-associated wound closure, and Transwell migration/invasion, with the most consistent effects observed in tumor-derived breast cancer cell lines, particularly MDA-MB-231. However, measurable responses were also detected in non-tumorigenic MCF-10A cells, indicating that the effects observed under the present experimental conditions are not strictly restricted to malignant breast cancer cells and therefore do not establish intrinsic tumor selectivity. Importantly, the MCF-10A response cannot be classified as a definitive off-target effect, because miR-548f-3p may also regulate biologically relevant target networks in non-malignant mammary epithelial cells.

An additional limitation relates to the use of a synthetic miRNA mimic. Although a concentration-matched non-targeting mimic and untreated cells were included as controls, these controls cannot fully exclude sequence- or concentration-dependent off-target effects. Moreover, mimic-induced miR-548f-3p levels were not calibrated to the endogenous physiological range, and AGO2/RISC loading and intracellular mimic stability were not directly assessed. Although the 24–72 h qRT-PCR time course demonstrated a transient post-transfection expression profile, this should not be interpreted as direct evidence of mimic stability or AGO2/RISC incorporation. Accordingly, the observed molecular and phenotypic changes should be interpreted as effects associated with miR-548f-3p mimic transfection under the conditions used, rather than as evidence of exclusively on-target activity. Future studies incorporating physiological dose calibration, formal dose–response analysis, AGO2-associated miRNA assessment, direct mimic-stability measurements, additional non-malignant and primary mammary epithelial models, and complementary sequence-specific controls will be required to define specificity and therapeutic selectivity more rigorously.

Interpretation of the migration- and invasion-associated assays also requires caution. Although 2% FBS was used in the wound-healing assay to reduce proliferation-associated wound closure and serum-free medium was used in the upper chamber of the Transwell assays, these conditions do not eliminate the potential contribution of apoptosis, altered viability, or cell-cycle effects. Because Mitomycin C treatment and normalization to viable input cell numbers were not performed, the reduced wound closure and Transwell cell counts cannot be interpreted as evidence of a migration- or invasion-specific mechanism.

Furthermore, the biological effects of miR-548f-3p should be interpreted within the broader context of the pleiotropic nature of miRNA-mediated regulation. Rather than acting through a single downstream effector, miRNAs typically modulate networks of target genes that collectively influence cellular phenotypes. Accordingly, the effects observed following miR-548f-3p restoration in TNBC cells are likely to reflect the coordinated regulation of multiple downstream targets rather than the exclusive involvement of ANP32E. In the present study, ANP32E was prioritized because of convergent evidence, including its TNBC-enriched expression, strong inverse association with miR-548f-3p in patient samples, predicted miR-548f-3p binding support, and reduced expression following miR-548f-3p restoration. Nevertheless, additional miR-548f-3p-responsive targets may contribute to the observed effects on apoptosis, cell-cycle progression, migration, and invasion. These phenotypic changes should therefore be interpreted as reflecting the broader regulatory activity of miR-548f-3p rather than as effects mediated exclusively by ANP32E.

Consistent with this interpretation, the present study does not establish a direct causal interaction between miR-548f-3p and ANP32E or demonstrate that ANP32E is the specific mediator of the observed cellular phenotypes. Instead, integrated in silico target prediction, structure-guided modeling, inverse clinical expression analysis, and post-transfection mRNA and protein measurements collectively identify ANP32E as an expression-responsive candidate downstream effector of miR-548f-3p. Restoration of miR-548f-3p was associated with reduced ANP32E expression in breast cancer cell lines, including the TNBC model MDA-MB-231; however, these findings should be regarded as supportive evidence for a putative regulatory relationship rather than definitive evidence of direct target engagement. Direct, site-dependent targeting of the ANP32E 3′UTR will require validation using wild-type and mutant reporter constructs, whereas dedicated ANP32E rescue experiments will be necessary to determine its relative contribution to the phenotypic effects of miR-548f-3p restoration. Future mechanistic studies examining ANP32E together with additional miR-548f-3p-responsive targets will therefore be important for defining the broader post-transcriptional regulatory network underlying the observed tumor-suppressive phenotypes.

Given the previously described role of ANP32E as a chromatin-associated regulator implicated in H2A.Z dynamics, E2F1-driven transcriptional programs, and cell-cycle progression, its modulation may plausibly contribute to a subset of the phenotypic effects observed after miR-548f-3p restoration [14,15,16,17,18,19,20,21]. Accordingly, the present findings should be interpreted within a broader post-transcriptional regulatory framework in which miR-548f-3p likely exerts its biological activity through the coordinated modulation of multiple oncogenic and cell-state-associated targets rather than through a single effector pathway.

Collectively, these findings support miR-548f-3p as a previously unrecognized tumor-suppressive miRNA candidate in TNBC and suggest that reduced miR-548f-3p expression may be associated with malignant phenotypes that are attenuated following mimic-mediated restoration. Our data support a model in which miR-548f-3p may modulate TNBC-associated cellular behaviors, at least in part, through post-transcriptional regulation of candidate downstream effectors such as *ANP32E*, although the mechanistic hierarchy among multiple predicted targets remains to be established. These observations align with emerging evidence supporting miRNA replacement strategies as a means of restoring dysregulated post-transcriptional regulatory circuits in cancer, as exemplified by preclinical studies of miR-22-3p delivery [11].

Despite these promising results, several limitations should be acknowledged. All functional experiments were conducted in vitro, and further studies will be required to evaluate miR-548f-3p delivery, stability, biodistribution, tumor penetration, and therapeutic tolerability in vivo—key challenges for miRNA-based therapeutics [26]. The protein-level assessment was additionally limited to a single biological experiment performed at the 48 h post-transfection endpoint. Accordingly, the Western blot and associated densitometric analysis should be considered descriptive and semi-quantitative, and neither the temporal profile nor statistically reproducible changes in ANP32E protein abundance can be established from the present data. Independent biological replication and protein time-course analysis will therefore be required for quantitative confirmation.

The clinical cohort was also modest in size, and the diagnostic and prognostic analyses should therefore be considered exploratory. Although higher miR-548f-3p expression was associated with a lower observed hazard of death, only 16 mortality events occurred, substantially limiting the scope for robust multivariable modeling. The relatively wide confidence interval around the hazard ratio and modest concordance index (0.651) further indicate limited prognostic precision and discrimination. Targeted sensitivity analyses adjusting for BMI, age, and both covariates jointly yielded comparable effect estimates; however, these analyses do not establish miR-548f-3p as an independent prognostic biomarker. Residual confounding by tumor stage, treatment, and other clinicopathological factors cannot be excluded.

The ROC findings likewise require cautious interpretation. Although miR-548f-3p showed an AUC of 1.000 with little apparent loss of discriminatory performance in repeated stratified five-fold cross-validation, the analysis was based on a relatively small, single-cohort dataset, and the complete separation between TNBC and non-TNBC samples may partly reflect cohort-specific characteristics and sampling variability. The ROC results should therefore be regarded as exploratory and hypothesis-generating rather than as evidence of established diagnostic performance. Internal cross-validation provides an assessment of within-cohort reproducibility but does not substitute for validation in larger, more diverse, and fully independent patient cohorts. In addition, the present MCF-10A findings should not be interpreted as evidence of normal-tissue safety, and therapeutic application would require demonstration of a favorable differential response between malignant and non-malignant cells.

Importantly, genetic rescue experiments were not performed, and therefore the extent to which ANP32E contributes to the phenotypic effects associated with miR-548f-3p restoration remains unresolved. Future studies using wild-type and mutant ANP32E 3′UTR reporter constructs will be required to determine whether miR-548f-3p directly and site-dependently targets the ANP32E 3′UTR, whereas ANP32E knockdown and rescue experiments will be necessary to define the functional contribution of ANP32E to the observed effects on apoptosis, cell-cycle progression, migration, and invasion. These studies will also help clarify the relative position of ANP32E within the broader, likely multi-target, post-transcriptional regulatory network of miR-548f-3p.

The upstream mechanisms responsible for miR-548f-3p downregulation, including potential genomic loss, epigenetic regulation such as DNA methylation, and altered transcriptional control, were not directly investigated and remain to be determined. Tumor-grade information was unavailable in the present cohort, and although patients were treatment-naïve at tissue collection, longitudinal treatment-response data were not available. Larger, independently assembled and more comprehensively annotated TNBC cohorts will therefore be required to further define the clinicopathological, prognostic, and potential predictive relevance of miR-548f-3p. Finally, in vivo studies will be necessary to evaluate the biological effects, therapeutic potential, selectivity, and safety of miR-548f-3p-based intervention strategies.

## 4. Material and Methods

### 4.1. Study Design

This study was designed as an integrative two-phase investigation combining computational prioritization with experimental assessment. In the in silico phase, miR-548 family members were initially evaluated using transcriptomic datasets, followed by gene-network analysis, target prediction, molecular docking, and single-cell RNA-seq interrogation to prioritize tumor-relevant miRNA–mRNA regulatory interactions in triple-negative breast cancer (TNBC). Based on these analyses, miR-548f-3p and *ANP32E* were selected as a candidate downstream relationship for further investigation.

**Figure 11:**
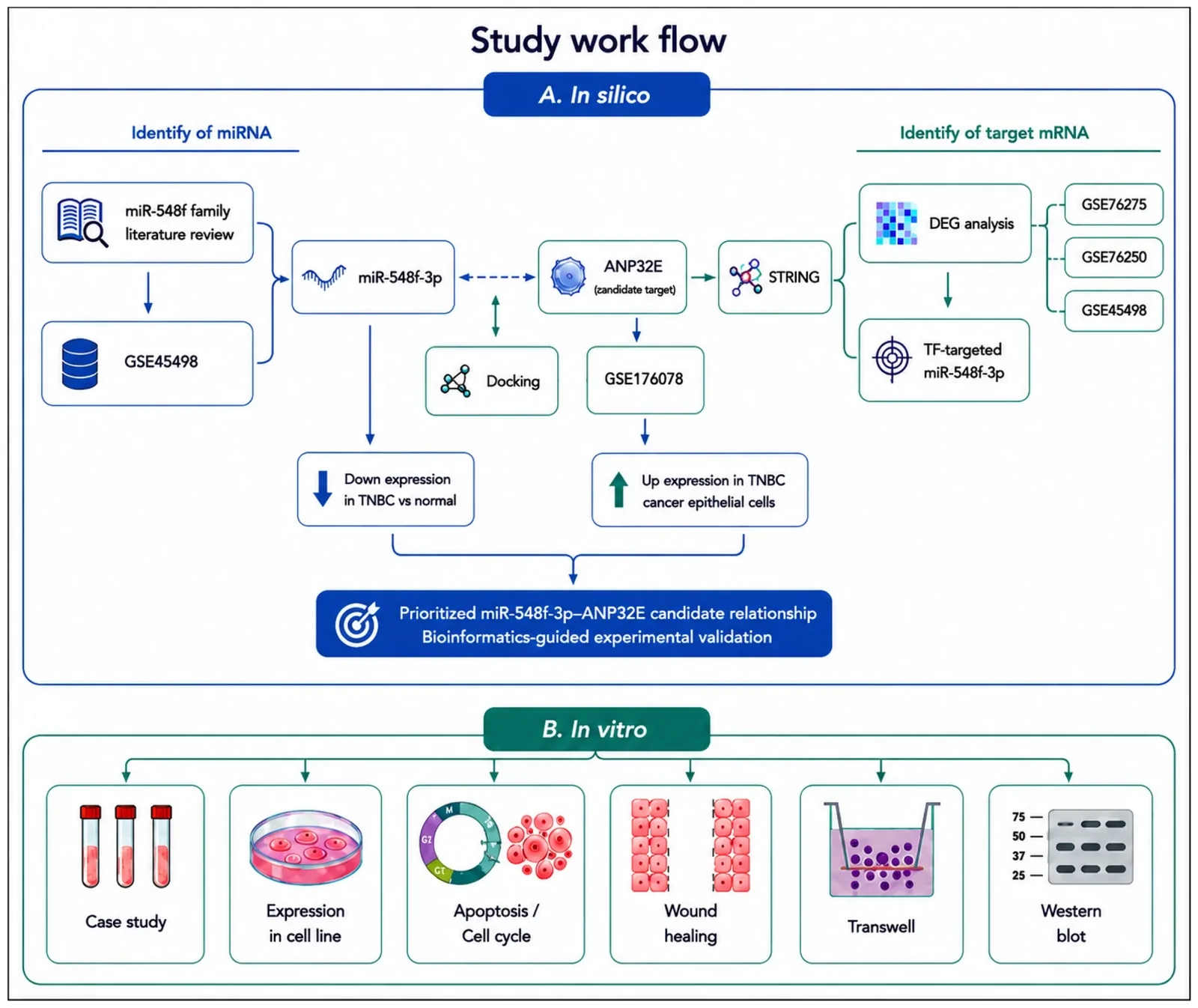
Workflow of the integrative computational and experimental approach used to investigate miR-548f-3p in TNBC and to prioritize *ANP32E* as a candidate downstream effector. The workflow integrates bioinformatics analyses (GEO datasets, target prediction, STRING, docking, and DEG analysis) with in vitro functional assays and assessment of ANP32E expression following miR-548f-3p restoration.

In the experimental phase, the biological relevance of miR-548f-3p was assessed using TNBC cell models and patient-derived tissue samples. miR-548f-3p mimic-based restoration was used to evaluate its effects on cell-cycle progression, apoptosis, migration, and invasion. The expression of miR-548f-3p and ANP32E was further examined at the transcript and protein levels to determine whether ANP32E expression was responsive to miR-548f-3p restoration (Figure 11).

### 4.2. Publicly Available Bioinformatics Resources

Publicly available bulk expression and single-cell RNA-seq datasets were retrieved from the Gene Expression Omnibus (GEO) database and used for integrative bioinformatics analyses. Three bulk expression datasets, GSE45498, GSE76250, and GSE76275, together with one single-cell RNA-seq dataset, GSE176078, were included.

For initial miRNA discovery, GSE45498 was used. This dataset comprises 165 primary TNBC tumors, 59 adjacent normal tissues, and 54 lymph node metastatic samples. This dataset is particularly suitable for miRNA analysis due to its well-annotated design and reliable detection of mature miRNA expression profiles. Expression profiling was performed using the NanoString nCounter platform, including GPL16231 for mature miRNA profiling and GPL16299 for cancer-associated mRNA expression profiling, enabling simultaneous assessment of both regulatory layers [27].

For mRNA-level differential expression analysis, GSE76250 was included. This dataset contains 165 TNBC samples and 33 paired normal breast tissues profiled using the Affymetrix Human Transcriptome Array 2.0 platform (GPL17586) [28]. GSE76250 was used to identify genes differentially expressed between TNBC tumors and normal breast tissues and to support transcriptome-level prioritization of candidate downstream effectors.

To compare TNBC-associated expression patterns with non-TNBC breast cancer, GSE76275 was also analyzed. GSE76275 is a GEO SuperSeries comprising 198 TNBC tumors and 67 non-TNBC breast cancer samples generated on the Affymetrix Human Genome U133 Plus 2.0 Array platform (GPL570) [29]. This dataset was used as an independent bulk transcriptomic resource to assess whether candidate genes showed TNBC-enriched expression relative to other breast cancer subtypes.

Cell-type-resolved expression patterns were interrogated using GSE176078, a single-cell RNA-seq dataset comprising 26 primary breast cancer samples, including 10 TNBC, 11 ER-positive, and 5 HER2-positive tumors, with matched bulk RNA-seq data available for 24 samples. Single-cell RNA sequencing was performed using the 10× Genomics Chromium platform, and sequencing was conducted on the Illumina NextSeq 500 system [30]. This dataset was used to evaluate the cellular distribution of candidate gene expression across malignant and non-malignant compartments and to validate bulk-derived findings at single-cell resolution.

All dataset characteristics, including accession numbers, sample composition, experimental platforms, and analytical purposes, are summarized in Table 4.

Sequences used for miRNA–mRNA interaction analyses were obtained from public nucleotide databases. The human *ANP32E* RefSeq transcript variant 5 sequence was retrieved from NCBI GenBank/RefSeq under accession NM_001280560.2, and the mature hsa-miR-548f-3p sequence was obtained from miRBase under accession MIMAT0005895. The corresponding *ANP32E* 3′ untranslated region (3′UTR) was extracted using the UCSC Genome Browser [31,32] based on the selected reference transcript and used for downstream miRNA-binding analyses.

### 4.3. Sample Collection

Primary invasive ductal carcinoma (IDC) tumor specimens and matched adjacent normal breast tissues were collected from patients undergoing surgical resection at Imam Khomeini Hospital, Tehran, Iran. Adjacent normal tissues were obtained from macroscopically tumor-free areas and were subsequently confirmed as histologically non-malignant by pathological examination. All patients were treatment-naïve at the time of sample collection and had not received chemotherapy, radiotherapy, endocrine therapy, or other systemic anticancer treatment before surgery.

A total of 60 pathologically confirmed breast cancer tumor specimens were included, comprising 30 triple-negative breast cancer (TNBC) tumors and 30 non-TNBC breast cancer tumors, together with their patient-matched adjacent normal tissues. TNBC status was defined by the absence of estrogen receptor (ER), progesterone receptor (PR), and human epidermal growth factor receptor 2 (HER2) expression/amplification according to routine pathological assessment. Non-TNBC specimens included breast tumors with positive ER, PR, and/or HER2 status. Following surgical excision, tissue samples were immediately snap-frozen in liquid nitrogen and stored at −80 °C until RNA and/or protein extraction.

Written informed consent was obtained from all participants before sample collection. All patients provided informed written consent, and the study protocol was approved by the Institutional Review Board and complied with the Declaration of Helsinki and the National Declaration of Ethical Principles (IR.MODARES.REC.1398.148).

### 4.4. Cell Culture

The human breast cancer cell lines MDA-MB-231 and MCF-7, representing TNBC and hormone receptor-positive breast cancer models, respectively, and the non-tumorigenic human mammary epithelial cell line MCF-10A were obtained from Bonyakhteh Research Center, Tehran, Iran. The MDA-MB-231, MCF-7, and MCF-10A cell lines were used as previously characterized breast cell models [33,34,35]. Cell line identity was confirmed by short tandem repeat (STR) profiling, and all cultures were confirmed to be mycoplasma-free before experimental use.

### 4.5. In Silico Analysis

#### 4.5.1. Computational Screening and Prioritization of Candidate miRNAs in TNBC

Expression matrices were downloaded from GEO and analyzed in R version 4.0.5 [36] using the limma package version 3.46.0 [37]. NanoString miRNA expression data were background-corrected, quantile-normalized, and annotated according to mature miRNA identifiers from miRBase. Differential expression analysis was performed using the empirical Bayes moderated-statistics framework implemented in limma, and *p* values were adjusted for multiple testing using the Benjamini–Hochberg false discovery rate (FDR) method. In the global analysis, miRNAs with |log_2_FC| ≥ 0.5 and an FDR-adjusted *p* value < 0.05 were classified as significantly differentially expressed.

The miR-548 family was selected a priori for focused investigation because it represents one of the largest miRNA families in the human genome and several of its members have been implicated in cancer-associated regulatory processes across different malignancies. The mature miR-548 family members included in the screening analysis are listed in Supplementary Table S1.

For initial miRNA-level screening, the GEO dataset GSE45498 was analyzed using mature miRNA expression data generated on the GPL16231 NanoString nCounter platform. This dataset includes primary TNBC tumors, adjacent normal breast tissues, and lymph node metastatic samples, enabling assessment of miRNA dysregulation across distinct TNBC-associated tissue states. The primary comparison of interest was primary TNBC tumors versus adjacent normal tissues. Additional pairwise comparisons were performed between lymph node metastases and adjacent normal tissues and between lymph node metastases and primary TNBC tumors to characterize broader patterns of miRNA deregulation.

Because the miR-548 family had been selected a priori for focused investigation, a secondary family-level screening step was subsequently performed. In this analysis, downregulated miR-548 family members with an FDR-adjusted *p* value < 0.05 were retained for candidate consideration even when the magnitude of differential expression did not meet the global |log_2_FC| ≥ 0.5 threshold. Such miRNAs were not classified as globally differentially expressed under the predefined criteria but were retained as family-focused candidates for hypothesis-driven prioritization. Final selection within this candidate set additionally considered the extent of prior characterization of individual miR-548 family members in breast cancer, particularly TNBC.

#### 4.5.2. Integrative Selection of Putative Downstream Target Genes

To identify putative downstream targets of miR-548f-3p, an integrative in silico workflow was applied by combining transcriptome-level differential expression analysis, miRNA target prediction, and network-based prioritization.

The mRNA expression component of GSE45498 (GPL16299) was initially inspected to maintain consistency with the miRNA discovery dataset. However, because this NanoString panel provides limited transcript coverage, two independent bulk mRNA datasets, GSE76250 (GPL17586) and GSE76275 (GPL570), were additionally analyzed to support transcriptome-wide identification of TNBC-associated candidate genes. GSE76250 was used to compare TNBC tumors with matched normal breast tissues, whereas GSE76275 was used to evaluate TNBC-associated expression patterns relative to non-TNBC breast cancer samples.

Differential expression analysis was performed using the limma package [37] with empirical Bayes-moderated linear modeling. When required, expression values were log_2_-transformed and normalized before analysis, and probe-level annotations were mapped to official gene symbols. For genes represented by multiple probes, the probe with the highest average expression was retained for downstream analysis. *p*-values were adjusted for multiple testing using the Benjamini–Hochberg false discovery rate method. Genes with an adjusted *p*-value < 0.05 and an absolute log_2_ fold change ≥ 1 were considered significantly differentially expressed. Given the reduced expression pattern of miR-548f-3p in TNBC, genes significantly upregulated in TNBC were retained as expression-compatible candidate targets for subsequent prioritization.

Putative mRNA targets of miR-548f-3p were retrieved using the multiMiR R package [38], which integrates curated experimentally supported miRNA–target interactions from databases including miRTarBase [39], TarBase [40], and miRecords [41], together with computationally predicted interactions from resources such as TargetScanHuman v7.2 [42]. Candidate genes were prioritized by intersecting miR-548f-3p target evidence obtained from validated and/or predicted miRNA–mRNA interaction resources with genes showing expression-compatible upregulation in the analyzed TNBC datasets. Cross-dataset concordance was subsequently assessed across the complementary transcriptomic datasets and was used as an additional prioritization criterion.

To further characterize regulatory relevance, overlapping candidate genes were annotated for transcription factor status using the Human Transcription Factors catalog described by Lambert et al. [43]. Protein–protein interaction analysis was performed using the STRING database [44], with Homo sapiens as the reference organism and a minimum required interaction score of 0.400 (medium confidence). Active interaction evidence sources included text mining, experiments, curated databases, co-expression, gene neighborhood, gene fusion, and gene co-occurrence. No additional first- or second-shell interactors were added, and the network was therefore restricted to the submitted query proteins. STRING-based functional connectivity was used as a supportive network-level prioritization criterion. Candidate genes were also examined using UALCAN [45] based on TCGA breast cancer data to assess subtype-associated expression patterns, with particular focus on TNBC-enriched expression.

Genes showing elevated expression in TNBC compared with normal breast tissues and/or non-TNBC breast cancer samples were considered to exhibit a TNBC-enriched expression pattern. Final candidate prioritization was based on sequential convergence across multiple analytical criteria rather than on a single composite numerical score. These criteria included TNBC-associated upregulation, miR-548f-3p target evidence, cross-dataset concordance, STRING-based functional connectivity, subtype-enriched expression in TCGA/UALCAN analyses, and supportive predicted binding characteristics. Candidates satisfying multiple independent criteria were considered high-priority downstream effectors and were advanced for subsequent analyses and experimental evaluation.

#### 4.5.3. Structural Modeling and Docking Analysis of Predicted miR-548f-3p–mRNA Interactions

Molecular docking is a computational approach widely used in drug discovery to predict molecular interactions between small molecules and biological targets [46]. Its application has also been extended to macromolecular interaction studies, including protein–protein [47] and RNA–RNA interactions [48], where it can provide structural hypotheses regarding the formation and stability of biomolecular complexes. In the present study, docking was incorporated as a complementary computational validation layer to assess the structural plausibility of predicted miRNA–mRNA interactions. This strategy was informed by the analytical framework reported by Sweetapple et al. [15], in which miRNA–mRNA duplex geometries were systematically evaluated in relation to multiple experimental platforms, including luciferase reporter assays, RNA affinity-based studies (RABS), and electrophoretic mobility shift assays (EMSA). Based on this rationale, we applied a comparable structural modeling approach to evaluate whether the predicted miR-548f-3p binding sites within candidate 3′UTRs could support geometrically plausible miRNA–mRNA duplex formation. Accordingly, docking analysis was used to strengthen target prioritization and provide supportive structural evidence for the proposed miR-548f-3p–candidate target interactions, while acknowledging that reporter-based assays remain the standard experimental method for definitive validation of direct 3′UTR targeting.

Using seed sequence predictions from TargetScan [42], we strategically truncated each 3′UTR with the aim of maintaining the canonical seed region, while avoiding disruption of pivotal secondary structure components. We then evaluated the folding and accessibility of the target sites using Vienna RNAfold with default parameters, a framework for examining RNA secondary structure [49], to confirm that the predicted miRNA binding regions were either unpaired or only weakly structured. Next, the miRNA–mRNA duplexes were modeled using MC-Fold, which generates minimum-energy duplex conformations, similar to co-folded interaction models [50,51].

Subsequently, the docking of these structures was performed with HNADOCK [52] a server designed to model three-dimensional interactions between RNA-RNA and RNA-DNA. Subsequently, docking of these structures was performed using HNADOCK [52], a server designed to model three-dimensional interactions between RNA–RNA and RNA–DNA complexes [48].

#### 4.5.4. Correlation Analysis with Cell-Cycle Regulatory Genes

Based on previous evidence linking *ANP32E* to cell-cycle regulation [21], correlation analysis was performed to evaluate the transcriptional association between *ANP32E* and representative *E2F/CCNE* cell-cycle regulatory genes in TNBC. Normalized expression values were extracted from the GSE76275 transcriptomic cohort. To minimize subtype-related confounding, the primary correlation analysis was conducted within TNBC samples only. *ANP32E* expression was correlated with the expression of *E2F1*, *CCNE1*, and *CCNE2*, which were selected as representative regulators of *E2F*-driven cell-cycle progression and G1/S transition.

Pairwise correlations were calculated using Spearman’s rank correlation coefficient. *p*-values were adjusted for multiple testing across the analyzed cell-cycle genes using the Benjamini–Hochberg false discovery rate method. Correlation coefficients and adjusted *p*-values were reported to assess the strength and statistical significance of *ANP32E*-associated cell-cycle gene co-expression patterns.

#### 4.5.5. Single-Cell Analysis of ANP32E Expression

To evaluate the cell-type distribution of ANP32E expression in triple-negative breast cancer (TNBC), single-cell RNA-seq data from GSE176078 (GPL18573) were analyzed using the processed expression matrix and accompanying metadata obtained from the GEO repository. Because the present analysis used the processed expression matrix and metadata provided with the original study, raw sequencing reads were not reprocessed and no additional upstream cell-level quality-control thresholds were imposed. Cell-type annotations as defined in the original dataset metadata were retained for downstream stratification. Prior to downstream analysis, the processed expression data and metadata were checked for consistency, including cell-barcode correspondence, availability of the genes of interest, and completeness of relevant sample, subtype, cell-type annotation, and available quality-control information. Importantly, miR-548f-3p was not represented as a quantifiable feature in the processed GSE176078 expression matrix and was therefore not evaluated at the single-cell level. This dataset includes primary breast tumor samples across multiple clinical subtypes; however, all analyses in the present study were restricted exclusively to TNBC-derived samples to ensure subtype-specific evaluation.

Cells were grouped into malignant epithelial, non-malignant epithelial (tumor microenvironment–derived epithelial cells), stromal, and immune compartments according to their assigned cell identities. Importantly, non-malignant epithelial cells refer to epithelial populations within the tumor microenvironment and do not represent healthy breast tissue.

ANP32E expression was evaluated across these cellular compartments using normalized single-cell expression values to assess enrichment in malignant and tumor-associated epithelial populations within TNBC. At the single-cell level, gene expression distributions were summarized using bubble (dot) plots, displaying mean log-normalized expression levels and the proportion of ANP32E-positive cells across annotated cell types.

For statistical inference, sample-level expression profiles were generated by aggregating normalized single-cell expression within each annotated cell type for each TNBC sample. Samples were screened for adequate cellular representation, and only samples containing the relevant matched cell populations were retained for paired statistical comparisons, thereby maintaining the patient/sample rather than individual cells as the unit of statistical inference. Differential expression between malignant epithelial cells and matched non-malignant epithelial compartments was assessed using paired Wilcoxon signed-rank tests. *p*-values were adjusted for multiple testing using the Benjamini–Hochberg false discovery rate (FDR) correction where applicable.

To further assess the association between ANP32E expression and the cycling malignant-cell phenotype, malignant epithelial cells were additionally stratified according to the original dataset annotations. Cells annotated as Cancer Cycling were compared with the remaining Cancer Epithelial population from the same TNBC samples. Sample-level ANP32E expression was compared between these matched malignant epithelial populations using a paired Wilcoxon signed-rank test, with FDR correction applied for multiple testing.

### 4.6. Clinical Validation and Statistical Analyses

#### 4.6.1. Clinical Tissue Samples and qRT-PCR Validation

A total of 120 breast tissue specimens were analyzed, including 30 triple-negative breast cancer (TNBC) tumors with 30 patient-matched adjacent normal tissues and 30 non-TNBC breast cancer tumors with 30 patient-matched adjacent normal tissues. Adjacent normal tissues from TNBC and non-TNBC patients were first evaluated separately. Because no significant difference was observed between the two adjacent normal groups for the analyzed expression markers, they were considered as a combined adjacent normal reference group for downstream comparative analyses, where appropriate. Accordingly, expression analyses were performed across TNBC tumors, non-TNBC tumors, and adjacent normal breast tissues.

Total RNA was extracted from tissue samples using TRIzol reagent (Invitrogen, Carlsbad, CA, USA) according to the manufacturer’s instructions. RNA concentration and purity were assessed using NanoDrop spectrophotometer (Thermo Fisher Scientific, Waltham, MA, USA), and samples with acceptable purity ratios were used for downstream analyses. The expression level of mature miR-548f-3p was quantified using a miRNA cDNA synthesis and quantitative real-time PCR kit (Bonyakhteh Co., Tehran, Iran), according to the manufacturer’s protocol. U6 small nuclear RNA was used as the internal reference control for miRNA normalization.

qPCR for miR-548f-3p was performed under the following thermal cycling conditions: initial denaturation at 95 °C for 15 min, followed by 40 cycles of 95 °C for 20 s and 60 °C for 60 s. Melting curve analysis was performed from 72 °C to 95 °C with increments of 0.3 °C per step to confirm amplification specificity. Relative miR-548f-3p expression was calculated using the 2^−ΔΔCt^ method.

In parallel, the mRNA expression of *ANP32E*, a putative downstream effector of miR-548f-3p, was quantified by real-time PCR using gene-specific primers. The primer sequences were as follows: *ANP32E* forward, 5′-TGATTTGGATACCACTCTGCAA-3′; *ANP32E* reverse, 5′-TCAAATACTTTTCCAGCTGCCT-3′. β-actin (*ACTB*) and glyceraldehyde-3-phosphate dehydrogenase (*GAPDH*) were used as endogenous reference genes for mRNA normalization. The primer sequences for the reference genes were as follows: *ACTB* forward, 5′-AGAGCTACGAGCTGCCTGAC-3′; *ACTB* reverse, 5′-AGCACTGTGTTGGCGTACAG-3′; *GAPDH* forward, 5′-GGTCACCAGGGCTGCTTTTA-3′; and *GAPDH* reverse, 5′-CCCGTTCTCAGCCATGTAGT-3′.

qPCR for mRNA targets was performed using the following thermal cycling protocol: initial denaturation at 95 °C for 15 min, followed by 38 cycles of 95 °C for 30 s, 58 °C for 30 s, and 72 °C for 30 s. Melting curve analysis was conducted from 72 °C to 95 °C with increments of 0.3 °C per step to confirm product specificity. Relative mRNA expression levels were calculated using the 2^−ΔΔCt^ method. All reactions were performed in triplicate, and no-template controls were included to monitor potential contamination.

#### 4.6.2. Correlation Analysis

The association between miR-548f-3p and *ANP32E* expression was assessed in TNBC tumor samples for which matched qRT-PCR measurements were available. Normalized relative expression values of miR-548f-3p and *ANP32E* were used for the analysis. Prior to correlation testing, data distribution and linearity were evaluated using Shapiro–Wilk test. Pearson’s correlation coefficient was applied when normality and linearity assumptions were satisfied; otherwise, Spearman’s rank correlation coefficient was used. Correlation coefficients, two-sided *p*-values, and 95% confidence intervals were reported.

#### 4.6.3. Clinicopathological Association Analysis

Within the TNBC cohort (*n* = 30), the association between miR-548f-3p expression and tumor stage was evaluated as an exploratory clinicopathological analysis. Tumor stage was treated as an ordinal variable (stages I–IV), and its association with normalized miR-548f-3p expression was assessed using Spearman’s rank correlation coefficient. Given the non-normal distribution of miR-548f-3p expression, differences in expression across the four tumor stages were additionally evaluated using the Kruskal–Wallis test. All tests were two-sided, and *p* values < 0.05 were considered statistically significant.

#### 4.6.4. Diagnostic Performance Analysis

The discriminatory performance of miR-548f-3p expression was evaluated using logistic regression and receiver operating characteristic (ROC) curve analyses. Normalized relative expression values of miR-548f-3p obtained from qRT-PCR were used as the predictor variable. A univariate logistic regression model was constructed to evaluate the ability of the investigated marker to discriminate triple-negative breast cancer (TNBC) from non-TNBC tumor samples. Receiver operating characteristic (ROC) analysis was subsequently performed based on this model to assess its subtype-discriminatory performance.

ROC curves were generated from the fitted logistic regression models or directly from miR-548f-3p expression values. The area under the ROC curve (AUC) was calculated to estimate discriminatory accuracy, and its 95% confidence interval was estimated using stratified bootstrap resampling with 10,000 repetitions. Optimal cutoff values were determined using Youden’s index, and the corresponding sensitivity and specificity values were reported. Exact binomial 95% confidence intervals were additionally calculated for sensitivity and specificity.

Internal stability was assessed using repeated stratified five-fold cross-validation with 100 repetitions in R, with classification thresholds determined from the training data and applied to held-out samples. Mean cross-validated AUC, sensitivity, specificity, and accuracy were calculated. The primary ROC analysis was performed using GraphPad Prism version 8.0 (GraphPad Software, San Diego, CA, USA), while the additional validation analyses were performed in R version 4.0.5 (R Foundation for Statistical Computing, Vienna, Austria).

All discriminatory performance analyses were considered exploratory and interpreted within the context of the study cohort, as internal cross-validation does not replace validation in an independent cohort.

#### 4.6.5. Exploratory Survival Analysis

The prognostic significance of miR-548f-3p expression was assessed using Kaplan–Meier survival analysis and Cox proportional hazards regression modeling. Overall survival was evaluated over a 5-year follow-up period. Patients were dichotomized into high- and low-expression groups according to the median miR-548f-3p expression value in the analyzed cohort. Survival curves were compared using the log-rank test. The association between miR-548f-3p expression group and overall survival was further assessed using univariable Cox proportional hazards regression. Hazard ratios (HRs) with corresponding 95% confidence intervals (CIs) and two-sided *p*-values were reported, and model discrimination was summarized using the concordance index.

To assess the potential influence of selected demographic covariates while limiting model complexity, additional parsimonious Cox models were fitted including BMI alone, age alone, and age and BMI jointly with the miR-548f-3p expression group. Age and BMI were modeled as continuous covariates. These analyses were considered sensitivity analyses rather than comprehensive multivariable prognostic models. The proportional hazards assumption for the BMI-adjusted Cox model was evaluated using Schoenfeld residuals.

### 4.7. In Vitro Cell-Based Experiments

MDA-MB-231 and MCF-7 cells were cultured in Dulbecco’s Modified Eagle Medium (DMEM; Gibco) supplemented with 10% heat-inactivated fetal bovine serum (FBS) and 1% penicillin–streptomycin. MCF-10A cells were maintained in DMEM/F12 medium supplemented with 5% horse serum, 20 ng/mL epidermal growth factor (EGF), 0.5 μg/mL hydrocortisone, 100 ng/mL cholera toxin, and 10 μg/mL insulin.

All cell lines were incubated at 37 °C in a humidified atmosphere containing 5% CO_2_ and were routinely subcultured at 70–80% confluence using 0.25% trypsin–EDTA. Cell cultures were routinely tested and confirmed to be free of mycoplasma contamination.

#### 4.7.1. miR-548f-3p Mimic Transfection

MDA-MB-231, MCF-7, and MCF-10A cells were seeded in six-well plates at densities of 2 × 10^5^, 2.5 × 10^5^, and 2.5 × 10^5^ cells per well, respectively. Cells were transfected when they reached approximately 50–70% confluence. Synthetic miR-548f-3p mimic and non-targeting negative control mimic (NC mimic; Miras Co., Tehran, Iran) were used at a final concentration of 50 nM in combination with Lipofectamine 2000 (Invitrogen, Carlsbad, CA, USA), according to the manufacturer’s instructions.

For each well, 100 pmol of miRNA mimic and 5 µL of Lipofectamine 2000 were diluted separately in Opti-MEM reduced-serum medium (Thermo Fisher Scientific, Waltham, MA, USA) and incubated for 5 min at room temperature. The diluted components were then mixed and incubated for an additional 15 min to allow transfection complex formation before being added to the cells. Culture medium was replaced with fresh complete medium 6 h after transfection. Untreated cells were included as a native control to monitor baseline cellular responses. The number of independent biological replicates for each experiment is specified in the corresponding figure legends.

#### 4.7.2. Assessment of Transfection Efficiency and Post-Transfection Gene Expression

To assess miR-548f-3p restoration and its effect on *ANP32E* expression, cells were harvested at 24, 48, and 72 h after transfection. Total RNA was extracted using TRIzol reagent (Invitrogen, USA) according to the manufacturer’s instructions. RNA concentration and purity were evaluated using a NanoDrop 2000 spectrophotometer (Thermo Fisher Scientific, USA), and samples with acceptable purity ratios were used for downstream analysis.

For miRNA expression analysis, RNA was reverse-transcribed using a miRNA cDNA synthesis kit (Bonyakhteh Co., Tehran, Iran), and miR-548f-3p expression was quantified by qPCR using SYBR Green Master Mix (Ampliqon A/S, Odense M, Denmark) on StepOnePlus Real-Time PCR System (Applied Biosystems, Foster City, CA, USA). U6 small nuclear RNA was used as the endogenous reference control for miR-548f-3p normalization.

For mRNA expression analysis, *ANP32E* expression was quantified by qPCR using gene-specific primers, and *GAPDH* was used as the endogenous reference gene for normalization. Relative expression levels of miR-548f-3p and *ANP32E* were calculated using the 2^−ΔΔCt^ method. All qPCR reactions were performed in triplicate, and no-template controls were included to monitor potential contamination.

### 4.8. Functional In Vitro Assays

#### 4.8.1. Cell Cycle Distribution and Apoptosis Analysis

For cell-cycle analysis, 5 × 10^5^ cells were harvested at 24 and 48 h post-transfection. Cells were washed with phosphate-buffered saline (PBS), fixed in 70% ethanol at −20 °C for at least 2 h, and subsequently washed to remove residual ethanol. Fixed cells were treated with RNase A (100 µg/mL) for 30 min at 37 °C and stained with propidium iodide (PI; 50 µg/mL). DNA content was analyzed using a FACSCalibur flow cytometer (BD Biosciences, San Jose, CA, USA). After exclusion of debris and cell doublets, the proportions of cells in the G_0_/G_1_, S, and G_2_/M phases were quantified using FlowJo software version 10.

Apoptosis was assessed using Annexin V–FITC/PI staining. Briefly, 1 × 10^5^ cells were harvested at 24 and 48 h post-transfection, washed with cold PBS, and resuspended in Annexin V binding buffer. Cells were then incubated with Annexin V–FITC and PI (BioLegend, San Diego, CA, USA) for 15 min at room temperature in the dark, according to the manufacturer’s instructions. Samples were analyzed by flow cytometry within 30–60 min after staining. Viable cells (Annexin V^−^/PI^−^), early apoptotic cells (Annexin V^+^/PI^−^), late apoptotic cells (Annexin V^+^/PI^+^), and necrotic cells (Annexin V^−^/PI^+^) were quantified based on quadrant gating of bivariate dot plots. Unless otherwise stated, experiments were performed in at least three independent biological replicates.

#### 4.8.2. Wound Healing (Scratch) Assay

Cell migration was evaluated using a wound-healing scratch assay. Cells were seeded in six-well plates at a density of 2 × 10^5^ cells/well and transfected with miR-548f-3p mimic or negative control mimic as described above. At 24 h post-transfection, cells were grown to near-confluence, and a uniform linear scratch was generated in each well using a sterile 200 µL pipette tip. Detached cells and debris were removed by washing with phosphate-buffered saline (PBS), and cells were maintained in culture medium containing 2% FBS to minimize proliferation-associated wound closure.

Images of the wound area were captured at 0, 24, and 48 h after scratching using an inverted microscope at 10× magnification (Nikon Eclipse TS2, Nikon Corporation, Tokyo, Japan). The same wound regions were imaged at each time point where possible. Wound area was quantified using ImageJ software (National Institutes of Health, Bethesda, MD, USA; https://imagej.net/ij/; accessed on 21 August 2026), and the percentage of wound closure was calculated as follows: wound closure (%) = [(wound area at 0 h − wound area at indicated time point)/wound area at 0 h] × 100. Data were obtained from at least three fields per well and three independent biological replicates, and statistical comparisons were performed as described in the statistical analysis section.

#### 4.8.3. Transwell Migration and Invasion Assays

Cell migration and invasion were assessed using Transwell chamber assays. At 48 h post-transfection, 1 × 10^5^ cells were resuspended in 200 µL serum-free medium and seeded into the upper chambers of Transwell inserts with 8 µm pore membranes (Corning Incorporated, Corning, NY, USA). The lower chambers were filled with 600 µL complete medium containing 10% fetal bovine serum (FBS) as a chemoattractant. For migration assays, cells were incubated for 24 h at 37 °C in a humidified atmosphere containing 5% CO_2_. After incubation, non-migrated cells on the upper surface of the membrane were gently removed using a cotton swab, whereas migrated cells on the lower surface were fixed with 4% paraformaldehyde and stained with 0.1% crystal violet. Migrated cells were quantified by counting five randomly selected microscopic fields per insert under a 20× objective.

For invasion assays, Transwell inserts were pre-coated with 50 µL Matrigel Basement Membrane Matrix (Corning, NY, USA) diluted 1:30 in cold serum-free medium. Coated inserts were incubated at 37 °C for 2 h to allow gel polymerization before cell seeding. Transfected cells were then seeded into the upper chambers as described above, and invasion was allowed to proceed for 48 h. non-invaded cells were removed from the upper surface of the membrane, and invaded cells on the lower surface were fixed, stained, and quantified using the same procedure described for the migration assay. Experiments were performed in three independent biological replicates, and statistical comparisons were conducted as described in the statistical analysis section.

#### 4.8.4. Western Blotting

At 48 h post-transfection, cells were washed with cold PBS and lysed in radioimmunoprecipitation assay (RIPA) buffer supplemented with a protease inhibitor cocktail. Cell lysates were centrifuged at 12,000× *g* for 15 min at 4 °C, and the supernatants were collected for protein analysis. Total protein concentration was determined using the bicinchoninic acid (BCA) protein assay kit (Thermo Fisher Scientific, USA), according to the manufacturer’s instructions.

Equal amounts of protein lysate (25 µg per sample) were mixed with protein loading buffer, denatured at 95 °C for 5 min, separated by sodium dodecyl sulfate–polyacrylamide gel electrophoresis (SDS-PAGE) on 10% gels, and transferred onto polyvinylidene difluoride (PVDF) membranes at 100 V for 90 min. Membranes were blocked with 5% non-fat milk in Tris-buffered saline containing 0.1% Tween-20 (TBST) for 1 h at room temperature and then incubated overnight at 4 °C with the following primary antibodies: anti-ANP32E rabbit polyclonal antibody (Abcam, Cambridge, UK; cat. no. ab5993; 1:2000), β-actin mouse monoclonal antibody (2A3; Santa Cruz Biotechnology, Dallas, TX, USA; cat. no sc-517582; 1:5000), and GAPDH mouse monoclonal antibody (6C5; Santa Cruz Biotechnology, cat. no. sc-32233; 1:5000).

After washing with TBST, membranes were incubated with the appropriate horseradish peroxidase (HRP)-conjugated secondary antibodies for 1 h at room temperature, including goat anti-mouse IgGκ-HRP (Santa Cruz Biotechnology, cat. no. sc-516102; 1:5000) and mouse anti-rabbit IgG-HRP (Santa Cruz Biotechnology, cat. no. sc-2357; 1:5000). Protein bands were visualized using an enhanced chemiluminescence (ECL) detection system, and band intensities were measured using ImageJ software (National Institutes of Health, Bethesda, MD, USA; https://imagej.net/ij/; accessed on 21 August 2026). Western blot analysis was performed at the 48 h post-transfection time point only. Both GAPDH and β-actin were evaluated as loading controls; however, β-actin was used for ANP32E densitometric normalization to minimize ambiguity associated with the relatively close apparent molecular masses of ANP32E and GAPDH. ANP32E band intensity was normalized to the corresponding β-actin signal. The available Western blot analysis represented a single biological experiment; therefore, the densitometric values were treated as descriptive, semi-quantitative measurements and were not subjected to inferential statistical analysis.

### 4.9. Statistical Analysis

Statistical analyses were performed using GraphPad Prism software version 8.0 [53]. Data distribution was assessed for normality using the Shapiro–Wilk test. Normally distributed quantitative data are presented as mean ± standard deviation (SD), whereas non-normally distributed data are presented as median with interquartile range (IQR), unless otherwise stated.

For comparisons between two independent groups, an unpaired two-tailed Student’s *t*-test or Welch’s *t*-test was used, depending on variance homogeneity. For comparisons among more than two groups, one-way analysis of variance (ANOVA) followed by Tukey’s or Dunnett’s multiple-comparisons test, as appropriate, was applied for normally distributed data, whereas the Kruskal–Wallis test was used for non-normally distributed data, followed by Dunn’s multiple-comparisons test where post hoc pairwise comparisons were performed. Dunnett’s test was used where multiple experimental groups were compared with a common control. Categorical variables were analyzed using the χ^2^ test or Fisher’s exact test, as appropriate.

The discriminatory performance of miR-548f-3p expression was assessed using logistic regression and receiver operating characteristic (ROC) curve analyses. The area under the ROC curve (AUC) was calculated to estimate discriminative ability, with its 95% confidence interval estimated using stratified bootstrap resampling with 10,000 repetitions. Optimal cutoff values were determined using Youden’s index [54]. Exact binomial 95% confidence intervals were calculated for sensitivity and specificity. Internal stability was further assessed using repeated stratified five-fold cross-validation with 100 repetitions in R, with classification thresholds determined from the training data and subsequently applied to held-out samples.

Overall survival was analyzed using the Kaplan–Meier method, and differences between survival curves were compared using the log-rank test. Univariable Cox proportional hazards regression was performed to estimate hazard ratios (HRs) and corresponding 95% CIs for the association between miR-548f-3p expression group and patient survival [55], and model discrimination was summarized using the concordance index. As targeted sensitivity analyses, additional parsimonious Cox models were fitted including BMI alone, age alone, and age and BMI jointly with the miR-548f-3p expression group. Age and BMI were modeled as continuous covariates. These analyses were considered sensitivity analyses rather than comprehensive multivariable prognostic models. The proportional hazards assumption for the BMI-adjusted Cox model was evaluated using Schoenfeld residuals. The correlation between miR-548f-3p and *ANP32E* expression was assessed using matched normalized qRT-PCR values from the same TNBC samples. Pearson’s correlation coefficient was used when normality and linearity assumptions were met; otherwise, Spearman’s rank correlation coefficient was applied. Within the TNBC cohort, the association between miR-548f-3p expression and ordinal tumor stage was assessed using Spearman’s rank correlation, and differences in expression across tumor stages were evaluated using the Kruskal–Wallis test.

For sample-level matched comparisons in the single-cell RNA-seq analysis, paired Wilcoxon signed-rank tests were used, with Benjamini–Hochberg false discovery rate (FDR) correction applied where appropriate. All statistical tests were two-sided, and *p* < 0.05 was considered statistically significant.

## 5. Conclusions

In conclusion, this study supports miR-548f-3p as a candidate tumor-suppressive miRNA in triple-negative breast cancer. miR-548f-3p was reduced in TNBC tissues and breast cancer cell models, and mimic-mediated restoration increased apoptosis, promoted G_0_/G_1_ accumulation, and reduced migration- and invasion-associated readouts. Integrative target prioritization, structural modeling, inverse expression analysis, and post-transfection mRNA and protein assessment support ANP32E as an expression-responsive candidate downstream effector of miR-548f-3p. However, the present findings do not establish direct 3′UTR-mediated targeting or demonstrate that ANP32E mediates the observed phenotypic effects. Direct, site-dependent targeting will require validation using wild-type and mutant 3′UTR reporter assays, whereas dedicated rescue experiments will be required to determine the functional contribution of ANP32E to miR-548f-3p-associated phenotypes. *RPIA* was also identified as a secondary candidate for future investigation consistent with the broader multi-target regulatory activity expected for miRNAs. The reduced expression of miR-548f-3p in TNBC and lymph node metastatic samples compared with normal tissues further supports its relevance to TNBC-associated disease states. Given the pleiotropic nature of miRNA-mediated regulation, the tumor-suppressive effects of miR-548f-3p are likely to involve coordinated modulation of multiple downstream targets. Together, these findings provide a rationale for further mechanistic and in vivo studies to define the biological and potential therapeutic relevance of miR-548f-3p in aggressive breast cancer.

## Figures and Tables

**Table 1:** FDR-significant downregulated miR-548 family members identified in the family-focused analysis of TNBC versus adjacent normal tissues (GSE45498).

miRNA	miRBase Accession	Sequence	log2FC	*p* Value	adj. *p* Value
hsa-miR-548g	MIMAT0005912	AAAACUGUAAUUACUUUUGUAC	−1.8097	6.23 × 10^−23^	2.93 × 10^−21^
hsa-miR-548a-5p	MIMAT0004803	AAAAGUAAUUGCGAGUUUUACC	−0.6324	4.73 × 10^−9^	3.91 × 10^−8^
hsa-miR-548i	MIMAT0005935	AAAAGUAAUUGCGGAUUUUGCC	−0.4994	1.20 × 10^−7^	8.07 × 10^−7^
hsa-miR-548a-3p	MIMAT0003251	CAAAACUGGCAAUUACUUUUGC	−0.3986	3.12 × 10^−6^	1.60 × 10^−5^
hsa-miR-548b-3p	MIMAT0003254	CAAGAACCUCAGUUGCUUUUGU	−0.3446	0.00111	0.00355
hsa-miR-548f-3p	MIMAT0005895	AAAAACUGUAAUUACUUUU	−0.3116	0.00847	0.02163
hsa-miR-548o	MIMAT0005919	CCAAAACUGCAGUUACUUUUGC	−0.274	0.01086	0.0268

Note: The predefined global differential-expression criterion was |log_2_FC| ≥ 0.5 together with FDR-adjusted *p* < 0.05. Because the miR-548 family was selected a priori for focused analysis, this table additionally reports downregulated family members with FDR-adjusted *p* < 0.05 that did not meet the global fold-change threshold. Such miRNAs were treated as family-focused candidates rather than as formally defined global differentially expressed miRNAs.

**Table 2:** Comparative prioritization of *ANP32E* and *RPIA* as predicted miR-548f-3p targets and candidate downstream effectors in TNBC.

Criterion	*ANP32E*	RPIA
TNBC-specific upregulation	Yes	Yes
miR-548f-3p target prediction	Yes	Yes
Docking support	Yes	Yes
Binding sites	2 sites	1 site
Seed match	8-mer	7–8-mer
TNBC functional evidence	Yes	Limited
Main biological role	Chromatin/cell cycle	Metabolism
Final priority	Primary	Secondary

Note: No weighted composite numerical score was assigned. Final prioritization was based on sequential convergence across the criteria shown, including transcriptomic concordance, target-prediction evidence, network support, subtype-associated expression, predicted binding characteristics, and prior TNBC-specific functional evidence.

**Table 3:** Demographic and clinicopathological characteristics of patients with TNBC and non-TNBC breast cancer.

Variable	Non-TNBC (*n* = 30)	TNBC (*n* = 30)	*p*-Value
Age (years, mean ± SD)	53.5 ± 10.9	49.3 ± 11.2	0.147
BMI (kg/m^2^, mean ± SD)	26.2 ± 5.3	29.5 ± 7.0	0.047
Cancer stage (I–IV)	I: 20.0%/II: 43.3%/III: 33.3%/IV: 3.4%	I: 16.7%/II: 23.3%/III: 53.3%/IV: 6.7%	0.307
Lymph node involvement	Positive 60%/Negative 40%	Positive 53%/Negative 47%	0.795
Distant metastasis	Absent 93.3%	Absent 90.0%	0.503
Menopausal status	Pre 36.7%/Post 63.3%	Pre 53.4%/Post 46.6%	0.191

Note: Continuous variables are presented as mean ± SD; categorical variables as *n* (%). *p*-values were calculated using Welch’s *t*-test for continuous variables and χ^2^ or Fisher’s exact test for categorical variables. Statistical significance was set at *p* < 0.05.

**Table 4:** GEO datasets included in the present study.

Analytical Use	Sample Composition	Platform	Data Type	Dataset
miRNA discovery and miRNA–mRNA prioritization	165 primary TNBC tumors, 59 adjacent normal tissues, and 54 lymph node metastatic samples	GPL16231/GPL16299	miRNA/mRNA NanoString	GSE45498
TNBC vs. normal differential expression	165 TNBC, 33 paired normal	GPL17586	bulk mRNA array	GSE76250
TNBC vs. non-TNBC comparison	198 TNBC, 67 non-TNBC	GPL570	bulk mRNA array	GSE76275
cell-type-resolved validation	26 patients (16 non-TNBC and 10 TNBC; 24 bulk samples)	10× Genomics/Illumina NextSeq 500	scRNA-seq	GSE176078

## Data Availability

The datasets used and/or analyzed during the current study are available from the corresponding author on reasonable request.

## Supplementary Materials

The following supporting information can be downloaded at: https://www.mdpi.com/article/10.3390/ijms27177589/s1. Refs. [56,57,58,59,60,61,62,18,63,64,65,66,67,68,69,70,71,72,73,19,74,75,76,77,78,79,80,81,82,83,84,85,86,87,88,89,90,91,92,93] are cited in the Supplementary Materials file.

## Abbreviations

TNBC: Triple-Negative Breast Cancer, ER: Estrogen Receptor, PR: Progesterone Receptor, HER2: Human Epidermal Growth Factor Receptor 2, miRNA: MicroRNA, siRNA: Small Interfering RNA, GEO: Gene Expression Omnibus, R: R Statistical Software, WGCNA: Weighted Gene Co-expression Network Analysis, mRNA: Messenger RNA, *ANP32E*: Acidic Nuclear Phosphoprotein 32 Family Member E, RefSeq: Reference Sequence, 3′UTR: 3′ Untranslated Region, PCA: Principal Component Analysis, FBS: Fetal Bovine Serum, DMEM: Dulbecco’s Modified Eagle Medium, EGF: Epidermal Growth Factor, PI: Propidium Iodide, FITC: Fluorescein Isothiocyanate, RIPA: Radio-Immunoprecipitation Assay, SDS-PAGE: Sodium Dodecyl Sulfate–Polyacrylamide Gel Electrophoresis, PVDF: Polyvinylidene Difluoride, TBST: Tris-Buffered Saline with Tween-20, HRP: Horseradish Peroxidase, ECL: Enhanced Chemiluminescence, SD: Standard Deviation, ANOVA: Analysis of Variance, ROC: Receiver Operating Characteristic, HR: Hazard Ratio, CI: Confidence Interval, NC: Negative Control, STR: Short Tandem Repeat, IDC: Invasive Ductal Carcinoma, U6: U6 small nuclear RNA (internal reference), *GAPDH*: Glyceraldehyde-3-Phosphate Dehydrogenase, MCF-10A: Non-tumorigenic Human Mammary Epithelial Cell Line, MCF-7: Luminal Breast Cancer Cell Line (ER/PR-positive), MDA-MB-231: Triple-Negative Breast Cancer Cell Line, AGO2: Argonaute 2, CLIP-seq: Crosslinking Immunoprecipitation Sequencing, TN: Tumor Necrosis, H2A.Z: Histone 2A variant Z, TRIzol: Tri-reagent for RNA extraction, Lipofectamine: Lipid-based transfection reagent, MC-Fold: RNA secondary structure modeling software, HNADOCK: RNA-RNA/RNA-DNA docking server, TargetScan: miRNA target prediction tool, RegRNA 2.0: Regulatory RNA analysis web tool, TCGA-BRCA: The Cancer Genome Atlas—Breast Cancer dataset, UALCAN: Online portal for TCGA data analysis. NCBI: National Center for Biotechnology Information, UCSC: University of California, Santa Cruz, HRs: hazard ratios, *GAPDH*: Glyceraldehyde-3-Phosphate Dehydrogenase, BCA: Bicinchoninic Acid, RABS: RNA affinity-based studies, EMSA: electrophoretic mobility shift assays, MFE: Minimum free energy.
