# Supplementary material for: Integrative Molecular Profiling of miR-548f-3p in Triple-Negative Breast Cancer Highlights ANP32E as a Candidate Downstream Effector
Source: Int J Mol Sci. 2026 Aug 25;27(17):7589. doi: 10.3390/ijms27177589 (PMC13566833; doi:10.3390/ijms27177589)
Supplement: Supplementary file 1 [file ijms-27-07589-s001.zip › ijms-4492951-supplementary.pdf]

Table S1. Cancer-related studies of MIR548 family members

MIR548 family member	Cancer type	Publication year(s)	Ref
miR-548	Gastric cancer	2023	[57]
miR-548a	Non-small cell lung cancer	2021	[58]
miR-548a-5p	Hepatocellular carcinoma	2016	[59]
miR-548a-3p	Colon	2022	[60]
miR-548b	Colorectal cancer	2020	[61]
miR-548b	Melanoma	2021	[62]
miR-548b	Oral tongue squamous cell carcinoma	2017	[63]
miR-548b-3p	Breast cancer	2020	[64]
miR-548b-3p	Lung cancer	2020	[65]
miR-548b-5p	Breast cancer	2022	[66]
miR-548c	Endometrial and ovarian cancer	2016	[67]
miR-548c-3p	Breast cancer	2020	[68]
miR-548c-3p	Colorectal cancer / 5-FU sensitivity	2023	[69]
miR-548c-3p	Glioma	2017	[70]
miR-548c-3p	Papillary thyroid carcinoma	2019	[71]
miR-548c-3p	Breast cancer	2026	[72]
miR-548c-5p	Colorectal cancer	2019	[73]
miR-548c-5p	Breast cancer	2018	[74]
miR-548d	Pancreatic cancer	2012	[75]
miR-548d-3p	Breast cancer	2016	[76]
miR-548d-3p	Gastric cancer	2020	[77]
miR-548d-3p	Osteosarcoma	2019	[78]
miR-548e	Ovarian cancer	2020	[79]
hsa-mir-548f-1 (MIR548F1)	Endometrial cancer	2022	[80]
hsa-mir-548f-1 (MIR548F1)	Hepatocellular carcinoma	2020	[81]
miR-548k	Breast cancer	2023	[82]
miR-548k	Breast cancer / multidrug resistance	2023	[83]
miR-548k	Esophageal squamous cell carcinoma	2018	[84]
miR-548i	Hepatocellular carcinoma	2021	[85]
miR-548i	Non-small cell lung cancer	2023	[86]
miR-548m	Breast cancer	2021	[87]
miR-548m	Non-Hodgkin B-cell lymphoma	2013	[88]
miR-548q	Nasopharyngeal carcinoma	2018	[89]
miR-548x-3p	Colorectal cancer	2025	[90]
miR-548aa	Colorectal cancer	2025	[91]
miR-548ag	Obesity-related endometrial cancer	2023	[92]
miR-548-3p	Breast cancer	2015	[93]
miR-548x	Glioblastoma	2020	[94]
miR-548o	Prostate cancer	2025	[95]
miR-548g	Gastric	2019	[96]

Abbreviations: 5-FU, 5-fluorouracil; MDR, multidrug resistance

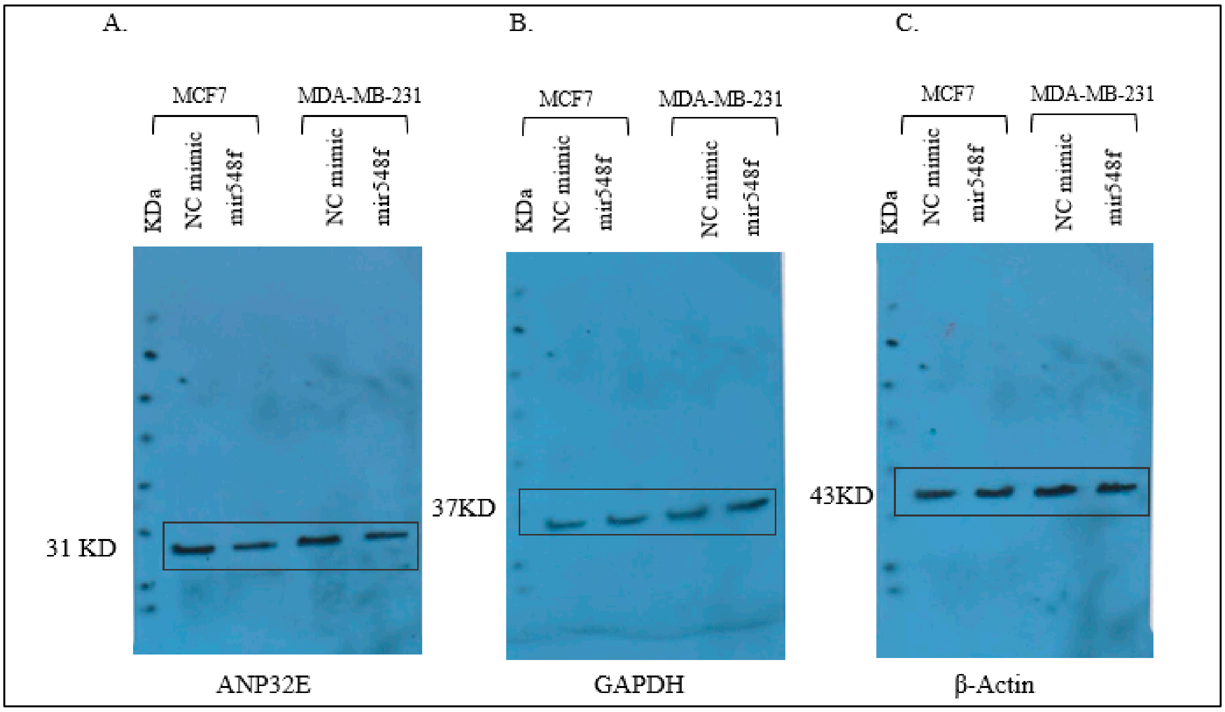

Figure S1. Full-length Western blot images corresponding to the descriptive protein-expression assessment presented in Figure 9. Full-length blot images from the single biological experiment performed following transfection of MCF-7 and MDA-MB-231 cells with the miR-548f-3p mimic or negative-control (NC) mimic are shown. **(A)** ANP32E (31 kDa), **(B)** GAPDH (37 kDa), and **(C)** β-actin (43 kDa). All samples were prepared and processed on the same day; however, ANP32E, GAPDH, and β-actin were resolved and transferred on three separate gels. Because ANP32E and the loading-control proteins were resolved on separate gels, the β-actin-normalized densitometric ratios reported in Figure 9 were treated strictly as descriptive, semi-quantitative measurements and were not subjected to inferential statistical analysis. The cropped regions presented in Figure 9 were derived from the corresponding blots shown here. Original, unprocessed full-length Western blot images have been provided separately to the journal as source data. NC, negative-control mimic.

.

References

57. Chen G, Shang J, Li M, Zhang H, Xu H. miR-548 predicts clinical prognosis and functions as a tumor promoter in gastric cancer. Clin Exp Med. 2023;23(5):1633–1639.
58. Zheng J, Li X, Cai C, Hong C, Zhang B. MicroRNA-32 and MicroRNA-548a Promote the Drug Sensitivity of Non-Small Cell Lung Cancer Cells to Cisplatin by Targeting ROBO1 and Inhibiting the Activation of Wnt/β-Catenin Axis. Cancer Manag Res. 2021;13:3005–3016.
59. Zhao G, Wang T, Huang QK, Pu M, Sun W, Zhang ZC, et al. MicroRNA-548a-5p promotes proliferation and inhibits apoptosis in hepatocellular carcinoma cells by targeting Tg737. World J Gastroenterol. 2016;22(23):5364–5373.
60. Wang M, Yang M, Deng B. miR-548a-3p Weakens the Tumorigenesis of Colon Cancer Through Targeting TPX2. Cancer Biother Radiopharm. 2022;37(10):917–926.
61. Xu Y, Zhong YD, Zhao XX. MiR-548b suppresses proliferative capacity of colorectal cancer by binding WNT2. Eur Rev Med Pharmacol Sci. 2020;24(20):10535–10541.
62. Feng XE. miR-548b Suppresses Melanoma Cell Growth, Migration, and Invasion by Negatively Regulating Its Target Gene HMGB1. Cancer Biother Radiopharm. 2021;36(2):189–201.
63. Berania I, Cardin GB, Clément I, Guertin L, Ayad T, Bissada E, et al. Four PTEN-targeting co-expressed miRNAs and ACTN4-targeting miR-548b are independent prognostic biomarkers in human squamous cell carcinoma of the oral tongue. Int J Cancer. 2017;141(11):2318–2328.
64. Sha MX, Huang XW, Yin Q. MiR-548b-3p inhibits proliferation and migration of breast cancer cells by targeting MDM2. Eur Rev Med Pharmacol Sci. 2020;24(6):3105–3112.
65. Wang Z, Wu X, Hou X, Zhao W, Yang C, Wan W, et al. miR-548b-3p functions as a tumor suppressor in lung cancer. Lasers Med Sci. 2020;35(4):833–839.
66. Záveský L, Jandáková E, Weinberger V, Minář L, Hanzíková V, Dušková D, et al. Small non-coding RNA profiling in breast cancer: plasma U6 snRNA, miR-451a and miR-548b-5p as novel diagnostic and prognostic biomarkers. Mol Biol Rep. 2022;49(3):1955–1971.
67. Sun X, Cui M, Zhang A, Tong L, Wang K, Li K, et al. MiR-548c impairs migration and invasion of endometrial and ovarian cancer cells via downregulation of Twist. J Exp Clin Cancer Res. 2016;35:10.
68. Tan PY, Wen LJ, Li HN, Chai SW. MiR-548c-3p inhibits the proliferation, migration and invasion of human breast cancer cell by targeting E2F3. Cytotechnology. 2020;72(5):751–761.
69. Khalili E, Afgar A, Rajabpour A, Aghaee-Bakhtiari SH, Jamialahmadi K, Teimoori-Toolabi L. MiR-548c-3p through suppressing Tyms and Abcg2 increases the sensitivity of colorectal cancer cells to 5-fluorouracil. Heliyon. 2023;9(11):e21775.
70. Lu J, Zhang M, Yang X, Cui T, Dai J. MicroRNA-548c-3p inhibits T98G glioma cell proliferation and migration by downregulating c-Myb. Oncol Lett. 2017;13(5):3866–3872.
71. Du Y, Zhu J, Chu BF, Yang YP, Zhang SL. MiR-548c-3p suppressed the progression of papillary thyroid carcinoma via inhibition of the HIF1α-mediated VEGF signaling pathway. Eur Rev Med Pharmacol Sci. 2019;23(15):6562–6569.
72. Bozkurt B, Ayan D, Bulut SM. Time-Dependent Loss of miR-548c-3p and Activation of E2F3/FOXM1 in Breast Cancer: In Vitro and TCGA-Based Evidence for a Post-Transcriptional Mechanism. Int J Mol Sci. 2026;27(2):1052.
73. Ge J, Li J, Na S, Wang P, Zhao G, Zhang X. miR-548c-5p inhibits colorectal cancer cell proliferation by targeting PGK1. J Cell Physiol. 2019;234(10):18872–18878.
74. Block I, Burton M, Sørensen KP, Andersen L, Larsen MJ, Bak M, et al. Association of miR-548c-5p, miR-7–5p, miR-210–3p, miR-128–3p with recurrence in systemically untreated breast cancer. Oncotarget. 2018;9(10):9030–9042.
75. Heyn H, Schreek S, Buurman R, Focken T, Schlegelberger B, Beger C. MicroRNA miR-548d is a superior regulator in pancreatic cancer. Pancreas. 2012;41(2):218–221.
76. Song Q, Song J, Wang Q, Ma Y, Sun N, Ma J, et al. miR-548d-3p/TP53BP2 axis regulates the proliferation and apoptosis of breast cancer cells. Cancer Med. 2016;5(2):315–324.
77. Liang H, Hu C, Lin X, He Z, Lin Z, Dai J. MiR-548d-3p Promotes Gastric Cancer by Targeting RSK4. Cancer Manag Res. 2020;12:13325–13337.

78. Chen J, Yan C, Yu H, Zhen S, Yuan Q. miR-548d-3p inhibits osteosarcoma by downregulating KRAS. Aging (Albany NY). 2019;11(14):5058–5069.
79. Zhang J, Quan LN, Meng Q, Wang HY, Wang J, Yu P, et al. MiR-548e Sponged by ZFAS1 Regulates Metastasis and Cisplatin Resistance of Ovarian Cancer by Targeting CXCR4 and let-7a/BCL-XL/S Signaling Axis. Mol Ther Nucleic Acids. 2020;20:621–638.
80. Ni L, Tang C, Wang Y, Wan J, Charles MG, Zhang Z, et al. Construction of a miRNA-Based Nomogram Model to Predict the Prognosis of Endometrial Cancer. J Pers Med. 2022;12(7):1154.
81. Li W, Kong X, Huang T, Shen L, Wu P, Chen QF. Bioinformatic analysis and in vitro validation of a five-microRNA signature as a prognostic biomarker of hepatocellular carcinoma. Ann Transl Med. 2020;8(21):1422.
82. Yadollahi-Farsani M, Amini-Farsani Z, Moayedi F, Khazaei N, Yaghoobi H. MiR-548k suppresses apoptosis in breast cancer cells by affecting PTEN/PI3K/AKT signaling pathway. IUBMB Life. 2023;75(2):97–116.
83. Saberiyan M, Ghasemi Z, Yaghoobi H. MiR-548 K regulatory effect on the ABCG2 gene expression in MDR breast cancer cells. Cancer Rep (Hoboken). 2023;6(6):e1816.
84. Chen Z, Lin J, Wu S, Xu C, Chen F, Huang Z. Up-regulated miR-548k promotes esophageal squamous cell carcinoma progression via targeting long noncoding RNA-LET. Exp Cell Res. 2018;362(1):90–101.
85. Jia C, Yao Z, Lin Z, Zhao L, Cai X, Chen S, et al. circNFATC3 sponges miR-548I acts as a ceRNA to protect NFATC3 itself and suppressed hepatocellular carcinoma progression. J Cell Physiol. 2021;236(2):1252–1269.
86. Yang W, Yao Y, Yang S, Ke Y. Circular RNA hsa_circ_0008003 promotes the progression of non-small-cell lung cancer by sponging miR-548I and regulating KPNA4 expression. Thorac Cancer. 2023;14(6):544–554.
87. Nor WFSBW, Chung I, Said NABM. MicroRNA-548m Suppresses Cell Migration and Invasion by Targeting Aryl Hydrocarbon Receptor in Breast Cancer Cells. Oncol Res. 2021;28(6):615–629.
88. Lwin T, Zhao X, Cheng F, Zhang X, Huang A, Shah B, et al. A microenvironment-mediated c-Myc/miR-548m/HDAC6 amplification loop in non-Hodgkin B cell lymphomas. J Clin Invest. 2013;123(11):4612–4626.
89. Zhuo X, Zhou W, Li D, Chang A, Wang Y, Wu Y, et al. Plasma microRNA expression signature involving miR-548q, miR-630 and miR-940 as biomarkers for nasopharyngeal carcinoma detection. Cancer Biomark. 2018;23(4):579–587.
90. Lei X, Qiu L, Chen Q, Liao L, Yu P, Wu W, et al. Exploring the regulatory mechanism of CCNA2 in colorectal cancer: Insights from multiomics and experimental analysis. J Biol Chem. 2025;301(8):110216.
91. Arabzadeh AA, Farzollahpour M, Seyedsadegi M, Pourfarzi F, Ghodsinezhad V, Bandehagh H, et al. Expression level of miR-548aa in tissue samples of patients with colorectal cancer. Mol Biol Rep. 2025;52(1):127.
92. Pang H, Wang J, Wei Q, Liu J, Chu X, Yuan C, et al. miR-548ag functions as an oncogene by suppressing MOB1B in the development of obesity-related endometrial cancer. Cancer Sci. 2023;114(4):1507–1518.
93. Shi Y, Qiu M, Wu Y, Hai L. MiR-548–3p functions as an anti-oncogenic regulator in breast cancer. Biomed Pharmacother. 2015;75:111–116.
94. Kalhori MR, Arefian E, Fallah Atanaki F, Kavousi K, Soleimani M. miR-548x and miR-4698 controlled cell proliferation by affecting the PI3K/AKT signaling pathway in Glioblastoma cell lines. Sci Rep. 2020;10(1):1558.
95. Wang C, Wei XP, Zhou C, Wang J, Zhang YF, He H, et al. Precise prediction of bone metastases and metastatic burden using exosomal miRNAs and radiomics: a multi-center study. J Transl Med. 2025;23:677.
96. Fang J, Hong H, Xue X, Zhu X, Jiang L, Qin M, et al. A novel circular RNA, circFAT1(e2), inhibits gastric cancer progression by targeting miR-548g in the cytoplasm and interacting with YBX1 in the nucleus. Cancer Lett. 2019;442:222–232.
